# Development of Kinase Inhibitors via Metal-Catalyzed C–H Arylation of 8-Alkyl-thiazolo[5,4-*f*]-quinazolin-9-ones Designed by Fragment-Growing Studies

**DOI:** 10.3390/molecules23092181

**Published:** 2018-08-29

**Authors:** Florence Couly, Marine Harari, Carole Dubouilh-Benard, Laetitia Bailly, Emilie Petit, Julien Diharce, Pascal Bonnet, Laurent Meijer, Corinne Fruit, Thierry Besson

**Affiliations:** 1 Normandie University, UNIROUEN, INSA Rouen, CNRS, COBRA UMR 6014, 76000 Rouen, France; florence.couly@insa-rouen.fr (F.C.); marine.harari@etu.univ-rouen.fr (M.H.); carole.dubouilh@univ-rouen.fr (C.D.-B.); laetitia.bailly@insa-rouen.fr (L.B.); emilie.petit@insa-rouen.fr (E.P.); 2 Institut de Chimie Organique et Analytique (ICOA), Université d’Orléans, UMR CNRS, 7311 BP 6759, 45067 Orléans CEDEX 2, France; julien.diharce@univ-orleans.fr (J.D.); pascal.bonnet@univ-orleans.fr (P.B.); 3 ManRos Therapeutics, Perharidy Peninsula, 29680 Roscoff, France; meijer@manros-therapeutics.com

**Keywords:** thiazolo[5,4-*f*]quinazolin-9(8*H*)-ones, microwave-assisted synthesis, C–H arylation, protein kinases, DYRK1A, CDK5, GSK-3, CLK1, CK1

## Abstract

Efficient metal catalyzed C–H arylation of 8-alkyl-thiazolo[5,4-*f*]-quinazolin-9-ones was explored for SAR studies. Application of this powerful chemical tool at the last stage of the synthesis of kinase inhibitors allowed the synthesis of arrays of molecules inspired by fragment-growing studies generated by molecular modeling calculations. Among the potentially active compounds designed through this strategy, **FC162** (**4c**) exhibits nanomolar IC_50_ values against some kinases, and is the best candidate for the development as a DYRK kinase inhibitor.

## 1. Introduction

The increasing presence of sulfur in organic compounds of interest in veterinary applications and in medicine has motivated our investment in research programs dealing with the chemical and pharmacological evaluation of fused thiazoles, mostly inspired by marine alkaloids [1,2,3,4,5]. In this context, the design and synthesis of sulfur-containing heterocycles able to inhibit the catalytic activity of kinases have been explored [6,7,8,9,10,11,12,13,14,15,16]. Phosphorylation of proteins by these enzymes is a universal mechanism used by cells to control major physiological phenomena, and many diseases are associated with abnormal kinase activities [17,18]. In the last decade, 43 kinase inhibitors, mostly tyrosine kinase inhibitors, have been approved by the US Food and Drug Administration (FDA) [19,20,21], mainly for cancer therapy. Nowadays, the field is rapidly expanding towards serine/threonine kinases, including for other therapeutic indications (e.g. neurodegenerative diseases) [22,23]. Our group is now focused on the regulation of dual-specificity tyrosine phosphorylation-regulated kinase 1A (DYRK1A), a conserved eukaryotic kinase that belongs to the DYRK family. This family is also comprising DYRK1B, DYRK2, DYRK3, and DYRK4 [24,25,26]. DYRK kinases belong to the CMGC group, which includes cyclin-dependent kinases (**C**DKs), mitogen-activated protein kinases (**M**AP kinases), glycogen synthase kinases (**G**SK), and Ccd2-like kinases (**C**LKs) [27].

**Scheme 1:**
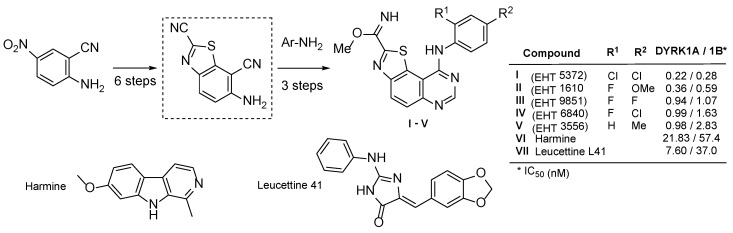
6-Aminobenzo[*d*]thiazole-2,7-dicarbonitrile, a versatile molecular platform for global chemistry strategy and structures of the five best DYRK1A/1B inhibitors. Harmine and Leucettine 41 (L41) are positive control inhibitors.

Recently, the kinase inhibitory potency of various *N*-aryl thiazolo[5,4-*f*]quinazolin-4-amines has been demonstrated, aiming at the improved treatment of Down syndrome (DS), early Alzheimer’s disease (AD), and cancers [12,13,14]. Specifically, a series of tricyclic aminopyrimidine derivatives was synthesized and evaluated on DYRK1A and DYRK1B. Five derivatives (EHT series) displayed single-digit nanomolar or subnanomolar IC_50_ values, and were quite specific towards the CMGC group (Scheme 1) [28].

At the same time, novel thiazolo[5,4-*f*]quinazolin-9(8*H*)-ones inspired by the EHT series [15,16] were synthesized by further modifications involving the development of a new synthetic methodology. The novel compounds were evaluated for their ability to modulate activity of kinases of the CGMC group, using in vitro enzyme functional assays. Switching the C9-aminosubstituted quinazolines (EHT series, Scheme 1) for the thiazolo[5,4-*f*]quinazolin-9(8*H*)-one analogues slightly reduced the activity, but the key effects of the carbimidate functional group in C2 were highlighted.

**Figure 1:**
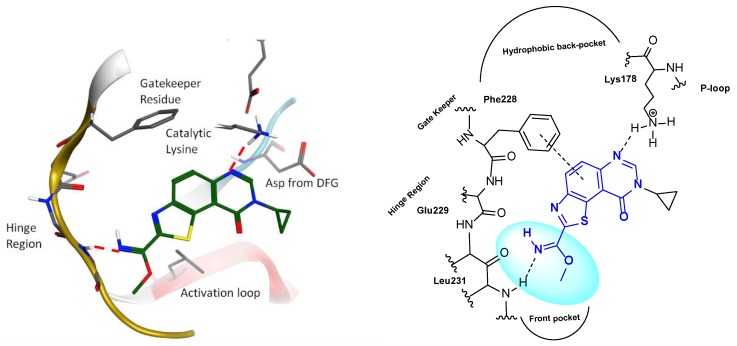
Results of molecular modeling studies (docking experiments) (**left**) and schematic representation (**right**) of the predicted binding modes of thiazolo[5,4-*f*]quinazolin-9(8*H*)-ones (Scheme 2, in DYRK1A).

Based on the results obtained with the crystal structure of DYRK2 in complex with EHT products [29] (Scheme 1, Figure 1), docking experiments and calculations were performed, and resulting models revealed that 9-oxo-inhibitors displayed binding modes identical to that of their 9-amino-congeners (Figure 1, left). As outlined in Figure 1, the methyl carbimidate function points to the hinge region with its nitrogen group.

On the basis of these new targeted thiazolo[5,4-*f*]quinazolin-9(8*H*)-ones, a fragment-growing approach was performed using a novel in silico tool that drills down through, to evaluate hundreds of thousands fragments extracted from co-crystallized kinase/inhibitor complexes. This innovative and appealing tool [30] generated more than a thousand novel compounds, sharing the same non-classical binding mode of the initial scaffold. Interestingly, addition of aromatic fragments on C2 seemed to increase the interaction with the hinge region (Scheme 3). Since the slight modification of the structure triggered by the introduction of an aryl residue upon the skeleton could have a major impact on its biological profile, a library of novel C2-arylated *N8*-alkyl thiazolo[5,4-*f*]quinazolin-9(8*H*)-ones was envisioned by addition of (hetero)-aromatic fragments (Scheme 3).

This article focuses on the design and synthesis of an array of 2-aryl-*N8*-alkylthiazolo[5,4-*f*]quinazolin-9(8*H*)-ones (series **4**) which were evaluated as potential kinase inhibitors. According to our recent experience in carbon–carbon bond formation [31,32], a regioselective C–H bond activation was planned to provide the corresponding C2-arylated valuable compounds (Scheme 4). Most of the syntheses described in this paper were achieved under microwave irradiation as a powerful alternative to traditional heating with economic and environmental benefits [33].

## 2. Results and Discussion

### 2.1. Chemistry

The target thiazolo[5,4-*f*]quinazolin-9(8*H*)-ones are substituted at *N8* position by an aliphatic chain and modified in C2 (Scheme 1) by an aromatic fragment, as predicted by the modeling studies. The envisioned retrosynthetic pathway is depicted in Scheme 4. Given that selective C–H arylation is an ideal strategy for late-stage functionalization, *N8*-benzylated-thiazolo-quinazolin-9(8*H*)-one (**1**) was chosen as model substrate.

The synthesis of the key intermediate **1** was previously reported in six steps (overall yield: 38%), starting from commercially available 5-nitroanthranilic acid under microwave irradiation [16]. The crucial steps were (a) reaction of an aromatic amine with 4,5-dichloro-1,2,3-dithiazolium chloride (Appel’s salt), a very versatile and useful sulfur-containing reactant [34]; (b) Cu-mediated cyclisation of the imino-1,2,3-dithiazole resulting from the nucleophilic attack of the amine allowed access to *N8*-benzyl-9-oxo-8,9-dihydrothiazolo[5,4-*f*]quinazoline-2-carbonitrile, which was decyanated (CN group hydrolysis and decarboxylation) by heating in HBr. This route has the advantage to be scalable, allowing an easy synthesis of the key intermediate **1** at a multigram scale (Scheme 5).

The most efficient conditions for the selective C2-H arylation of thiazolo[5,4-*f*]quinazolin-9(8*H*)-one **1** with aryl halides were previously determined [31,32]. The starting thiazolo[5,4-*f*]quinazolin-9(8*H*)-one **1** was heated in a sealed tube at 120 °C for 10 min in the presence of copper iodide (CuI, 1.0 equiv) and 1,8-diazabicyclo[5.4.0]undec-7-ene (DBU, 2.0 equiv) as a base, in dry DMF. After addition of Pd(OAc)_2_ (10 mol%) and the appropriate aryl halide (2.0 equiv), the reaction mixture was heated again at 120 °C for 5 h (Scheme 6, Table 1).

For the SAR studies, aryl iodides and bromides screened as coupling partners were mainly inspired by the Topliss tree [35], in order to maximize the chances of synthesizing the most potent compounds of series **2** as early as possible (Scheme 6, Table 1). Extending aryl partners to hetero-aryl halides allowed the synthesis of series **3** compounds in moderate yields. It should be noted that low yields primarily observed in the synthesis of **3a** (<20%) was explained by difficult work-up and purification. A better yield of 43% was obtained by using 1,5,7-triazabicyclo[4.4.0]dec-5-ene (TBD) [36] as a base instead of DBU.

The inhibitory potency of series **2** and **3** was evaluated according to standard methods [15,16] on a panel of kinases (for details see kinase profiling paragraph). Among the thiazolo[5,4-*f*]quinazolin-9(8*H*)-ones tested, only two molecules of series **3** (**3a** and **3b**) exhibited micromolar IC_50_ values against kinases CLK1 and GSK3, and nanomolar range inhibition against DYRK1A (see Table 1 and Scheme 6).

**Scheme 2:**
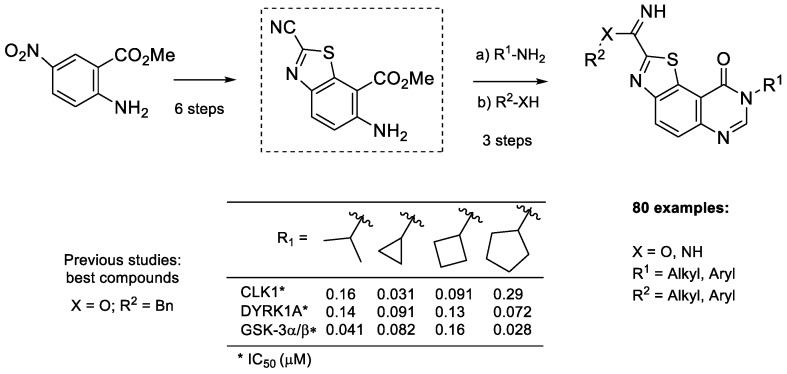
Schematic access to thiazolo[5,4-*f*]quinazolin-9(8*H*)-ones, a family of multitarget kinase inhibitors.

**Scheme 3:**
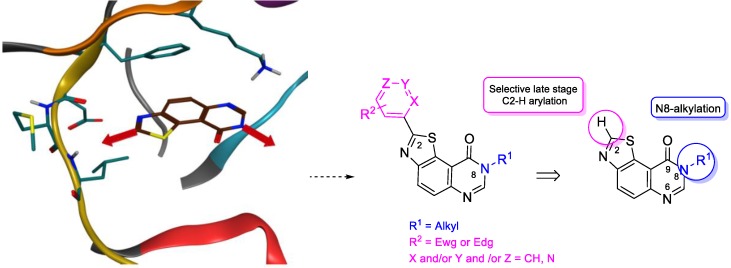
Fragment-growing anchor points envisioned on the thiazolo[5,4-*f*]quinazolin-9(8*H*)-one scaffold (**left**). Schematic representation of the predicted binding modes and retrosynthetic route to C2-arylated derivatives suggested via C–H arylation of the former C2-H thiazole precursor (**right**).

**Scheme 4:**
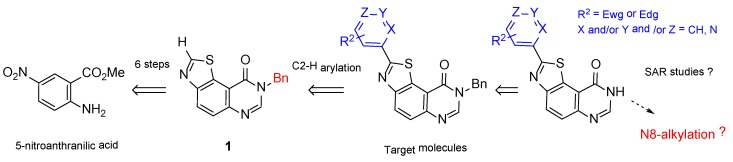
Retrosynthetic pathway envisioned.

**Scheme 5:**
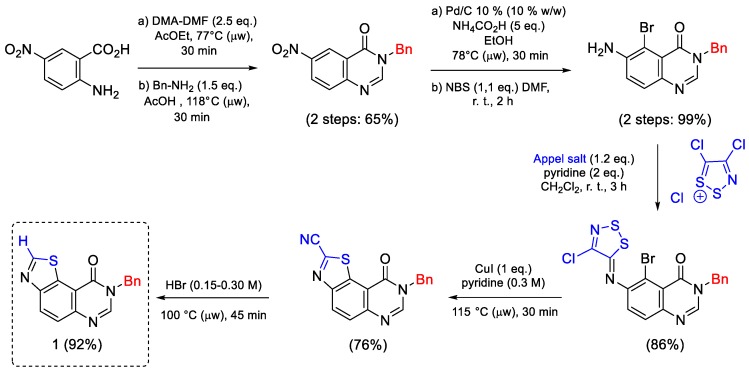
Six step synthesis of the key *N8*-benzylthiazolo[5,4-*f*]quinazoline (**1**) from 5-nitroanthranilic acid [16].

Taking these preliminary results into account, series **4** was designed by keeping the 3-pyridinyl moiety in position C2, and modifying the alkyl substituents in position *N8* of the thiazolo[5,4-*f*]quinazolin-9(8*H*)-ones. The envisioned modifications depicted in Scheme 7 were inspired by previous work on carbimidate derivatives described in Scheme 2 [15,16].

**Scheme 6:**
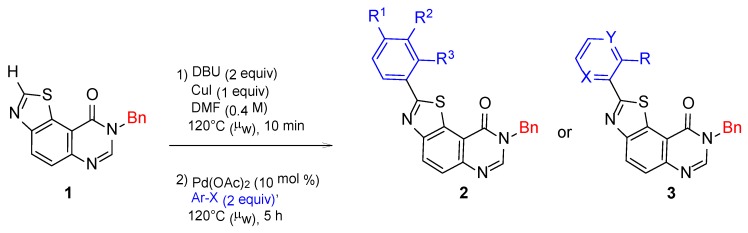
Selective C2-H arylation of **1** with aryl halides.

**Scheme 7:**
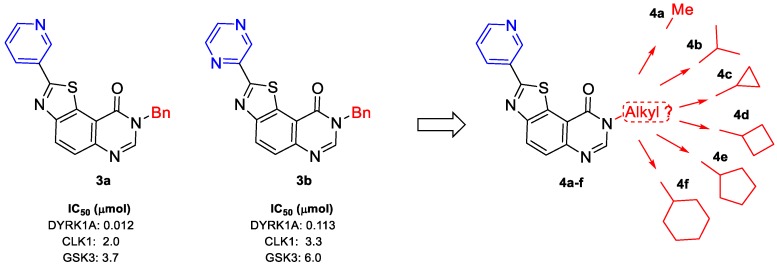
The two kinase active derivatives **3a** and **3b** and new compounds (series **4a**–**f**) suggested by the first active products.

**Scheme 8:**
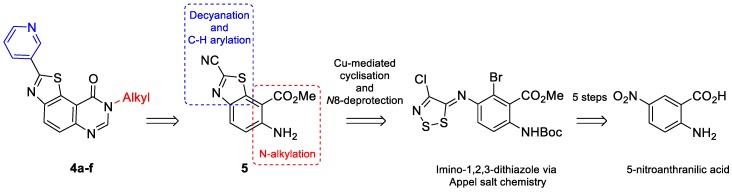
Retrosynthetic route of series **4** products using compound **5** as intermediate.

The target molecules (series **4**) were 2-(pyridin-3-yl)thiazolo[5,4-*f*]quinazolin-9(8*H*)-ones, substituted in position *N8* by an aliphatic fragment (Scheme 1). Alkylation of the corresponding debenzylated compound **3** (Scheme 6) was initially envisioned. Usual methods for the cleavage of the benzyl group [31] of compound **3a** led to the expected compound in yields up to 42%. Nevertheless, this *N8* deprotected derivative was found to be insoluble in most organic solvents, therefore discarding the alkylation pathway. Finally, the target molecules (series **4**) were synthesized via the polyfunctionalized methyl 6-amino-2-cyanobenzo[*d*]thiazole-7-carboxylate (**5**), a versatile precursor already described in previous studies [16]. Here, again, the key step in the synthesis of **5** involves the sulfur-rich Appel’s salt, and cyclization of the intermediate imino-1,2,3-dithiazole which was transformed into the target benzothiazole (Scheme 8). In this pathway, the quinazolinone part was formed at the last stage of the synthesis.

**Scheme 9:**
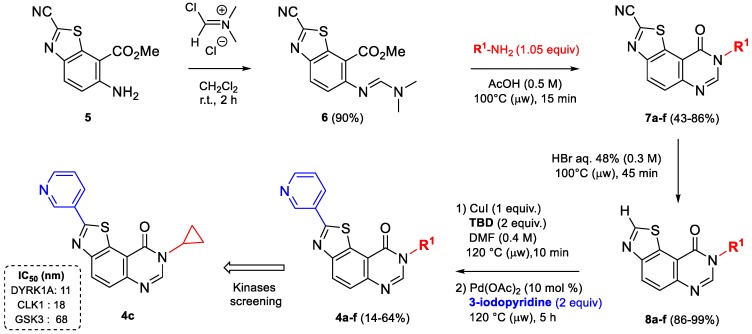
Synthesis of series **7a**–**f**, **8a**–**f**, and **4a**–**f**.

Treatment of aminoester **5** with 1.5 equivalents of Vilsmeier–Haack reagent in dichloromethane at room temperature gave (*E*)-methyl 2-cyano-6-([(dimethylamino)methylene]amino)benzo[*d*]-thiazole-7-carboxylate (**6**) in excellent yield (90%). This formimidamide was heated at 100 °C under microwave irradiation in the presence of 1.05 equivalents of appropriate amines, in acetic acid. After an irradiation time of 15 min, the corresponding *N8*-substituted-9-oxo-8,9-dihydrothiazolo[5,4-*f*]quinazoline-2-carbonitriles (series **7a**–**f**) were obtained in moderate to good yields (43–86%) [15] (Scheme 9, Table 2). Access to C2-H derivatives (series **8a**–**f**) was finally obtained by heating the corresponding precursors in HBr in sealed vials, under microwave irradiation.

**Scheme 10:**
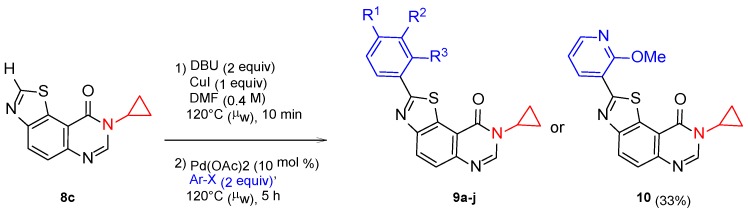
Synthetic route to series **9a**–**j** and **10** (R, X, and Y).

After the late-stage hetero-arylation, the small library of compounds was evaluated for their kinase inhibitory properties. As described for series **2a**–**f** and **3a**–**c**, the kinase profiling of series **4a**–**f** (for details see kinase profiling paragraph) highlighted the cyclopropyl derivative **4c**, which exhibits noteworthy activity against kinases with nanomolar IC_50_ values for DYRK1A, CLK1 and GSK3 (Scheme 9). Substituting 2-(pyridin-3-yl)thiazolo[5,4-*f*]quinazolin-9(8*H*)-one with alkyl groups, such as methyl, *iso*-propyl, or cycloakyl containing at least 4 carbons, resulted in complete loss of activity (see Table 2). Following this result, a new series, **9a**–**j**, was envisioned; by analogy with the first series **2a**–**j,** the palladium-catalyzed CH-arylation was applied to **8c**, and afforded series **9a**–**j** and compound **10** in moderate to good yields (Scheme 10 and Table 3) [32].

Microwave-assisted regioselective C–H bond arylation of the thiazolo[5,4-*f*]quinazolin-9(8*H*)-one skeleton thus provides an efficient and simplified route towards these valuable sulfur-containing bioactive heterocycles.

### 2.2. Kinase Profiling

Products of series **2a**–**j**, **3a**–**c**, **4a**–**f**, **9a**–**i**, and **10** were evaluated in five different in vitro kinase assays: CDK5/p25, CK1δ/ε (casein kinase 1), GSK-3α/β, DYRK1A (dual-specificity, tyrosine phosphorylation regulated kinase), and CLK1 [37,38,39].

All compounds were first tested at a final concentration of 10 μM. Compounds showing less than 50% inhibition were considered as inactive (IC_50_ >10 μM). Compounds displaying more than 50% inhibition at 10 μM were next tested over a wide range of concentrations (usually from 0.01 to 10 μM), and IC_50_ values were determined from the dose–response curves (Sigma-Plot). Harmine (Table 4), a β-carboline alkaloid (Scheme 1) known to inhibit DYRK1A [40], was used as a positive control.

Results provided in Table 4 demonstrate that none of the thiazolo[5,4-*f*]quinazolin-9(8*H*)-ones synthesized in series **2a**–**j** and **9a**–**i** showed significant inhibitory activity against the set of five kinases.

In series **4a**–**f**, only 8-cyclopropyl-2-(pyridin-3-yl)thiazolo[5,4-*f*]quinazolin-9(8*H*)-one (**4c**) (also called **FC162**) exhibited nanomolar IC_50_ values (11, 18, and 68 nM against DYRK1A, CLK1, and GSK3, respectively).

In series **3a**–**c**, two molecules exhibited nanomolar to submicromolar IC_50_ values against DYRK1A (IC_50_: 11 nM and 133 nM for **3a** and **3b**, respectively). It is remarkable that these two derivatives were poor inhibitors of CLK1 and GSK3 compared with **4c**, suggesting a high selectivity for DYRK1A. Except for kinase GSK3, the inhibition profile of **4c** for CLK1 and DYRK1A was close to that of Harmine.

**Figure 2:**
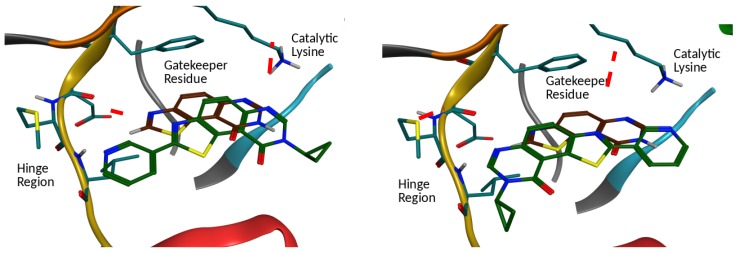
Predicted binding mode of **FC162** by docking calculation. **Left**, first predicted binding mode (green), with the same orientation of the skeleton, but slightly shifted. **Right**, second predicted binding mode, in which the skeleton is flipped compared to its initial placement (in brown).

Docking calculations were next performed in order to predict the molecular interactions of **FC162** with DYRK1A. Two main binding modes (Figure 2) were obtained. The first one maintained the initial position of the thiazolo[5,4-*f*]quinazolin-9(8*H*)-one skeleton, in which an interaction with the catalytic lysine is retrieved. The pyridine moiety interacts with the hinge region by forming a hydrogen bond with the backbone NH of Leu231. The second possible binding mode showed a complete flip of the molecule in the cavity. Indeed, the pyridine moiety interacted with the catalytic lysine, while the thiazolo[5,4-*f*]quinazolin-9(8*H*)-one skeleton interacted with the hinge region through hydrogen bond. The docking score of the two poses was quite similar, thus, both binding modes are equally possible for this compound.

**Figure 3:**
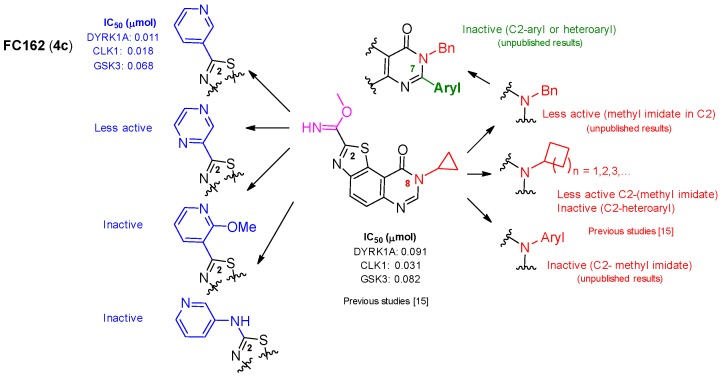
Comparative study of the activity of **FC162** (**4c**) with related molecules.

The SAR study revealed that **3a** and **4c** with a 3-pyridinyl group in position 2 had a higher activity than the series of phenylated derivatives **2** and **9**. These results are notably in agreement with the fragment-growing experiments, which suggested replacement of the imidate group by a more stable heteroaromatic substituent (Figure 3). The fact that **3c** and **10** were inactive demonstrates the importance of a free 3-pyridinyl group in C2, and may suggest that the planarity between the pyridinyl group and the tricyclic scaffold, broken with an ortho substituent on the pyridinyl group, is compulsory for DYRK inhibitory activity.

Other studies are currently in progress, in order to optimize interactions with hinge amino acid residues, and aiming to improve the affinity and selectivity of compounds for the targeted CLK1 and DYRK kinases.

## 3. Material and Methods

### 3.1. General Information 

All reagents were purchased from commercial suppliers and were used without further purification, except for DMF, which was stored under argon and activated molecular sieves. All reactions were monitored by thin-layer chromatography with silica gel 60 F254 (Merck Ltd., KGaA, Darmstadt, Germany) precoated aluminium plates (0.25 mm). Visualization was performed with a UV light at wavelengths of 254 nm. Purifications were conducted with a flash column chromatography system (Puriflash) equipped with a dual UV/vis spectrophotometer (200–600 nm), a fraction collector (176 tubes), a dual piston pump (1 to 200 mL/min, Pmax = 15 bar), which allowed quaternary gradients, and an additional inlet for air purge (Interchim, Montluçon, France). Melting points of solid compounds were measured with a SMP3 Melting Point instrument (STUART, Bibby Scientific Ltd., Roissy, France) with a precision of 1.5 °C. IR spectra were recorded with a Spectrum 100 Series FTIR spectrometer (PerkinElmer, Villebon S/Yvette, France). Liquids and solids were investigated with a single-reflection attenuated total reflectance (ATR) accessory; the absorption bands are given in cm^−1^. NMR spectra (^1^H and ^13^C) were acquired at 295 K using an AVANCE 300 MHz spectrometer (Bruker, Wissembourg, France) at 300 and 75.4 MHz, using TMS as an internal standard. Coupling constants *J* are in Hz, and chemical shifts are given in ppm. Signals in ^13^C spectra were assigned based on the result of ^13^CDEPT135 experiments (see Supplementary Materials). Mass spectrometry was performed by the Mass Spectrometry Laboratory of the University of Rouen. The mass spectra (ESI, EI, and field desorption (FD)) were recorded with an LCP 1er XR spectrometer (WATERS, Guyancourt, France). Microwave-assisted reactions were carried out in sealed tubes with a Biotage Initiator microwave synthesis instrument, and temperatures were measured by IR-sensor (Biotage, Uppsala, Sweden). Time indicated in the various protocols is the time measured when the mixtures were at the programmed temperature. The purity of all tested compounds was determined by chromatographic analysis performed at 25 °C on Ultimate 3000 (Thermo Scientific, Les Ulis, France) with a quaternary pump equipped with a photodiode array detector (DAD) managed at 254 nm. Column was a Luna C18 (150 mm × 4.6 mm; 3 μm particle size) provided by Phenomenex (Le Pecq, France). The mobile phase was water (A) and acetonitrile (B) (*v*/*v*); starting condition is 90% A and 10% B, in which the solvent B changed to 10% to 90% in 4% by min. Flow rate was 0.5 mL/min, and 5 µL were injected. The percentage of purity of all products was more than 98%.

### 3.2. Chemistry

Compounds **1** and **7a**–**f** were described in ref [15]; compounds **2a**–**i** and **3a**,**b** were described in ref [31]; compounds **2j**, **3c**, **4c**, **9j**, and **10** were described in ref [32]; The new products **8a**–**f** and **4a**, **4b** and **4d**–**f** are described below. The lead molecule **FC162** (**4a**) is described again. 

#### 3.2.1. General Procedure for the Synthesis of 8-Alkyl-thiazolo[5,4-*f*]quinazolin-9(8*H*)-one (**8a**–**f**) from 8-Alkyl-9-oxo-8,9-dihydrothiazolo[5,4-*f*]quinazoline-2-carbonitrile (**7a**–**f**)

In a 2–6 mL sealed tube, a suspension of the corresponding carbonitrile-bearing derivative **7a**–**f** (0.60 mmol, 1 equiv) and hydrobromic acid (48% in water) (2 mL, 0.3 M) was irradiated under microwave for 45–90 min at 100 °C. After cooling, the resulting solution was diluted with dichloromethane, and neutralized with a saturated aqueous solution of NaHCO_3_ and with solid Na_2_CO_3_ (until pH 8–9). The organic layer was then dried over Na_2_SO_4_, and concentrated under reduced pressure to provide the corresponding product **8a**–**f**.

*8-Methylthiazolo[5,4-f]quinazolin-9(8H)-one* (**8a**): Reaction time: 55 min; Yield: 98%; 112 mg; white solid; R*_f_*: 0.47 (DCM/MeOH, 95:5; *v*/*v*); mp 221–224 °C; IR (neat) ν_max_ 3308, 3149, 3077, 1902, 1698, 1664, 1589, 1386 cm^−1^; ^1^H-NMR (CDCl_3_, 25 °C, 300 MHz): *δ_H_* 9.22 (1H, s, H_2_), 8.48 (1H, d, *J* = 8.8 Hz, H_4_), 8.20 (1H, s, H_7_), 7.87 (1H, d, *J* = 8.8 Hz, H_5_), 3.73 (3H, s, CH_3_); ^13^C{^1^H}-NMR (CDCl_3_, 25 °C, 75.4 MHz): *δ_C_* 160.2 (C), 157.8 (CH), 152.5 (C), 147.1 (C), 146.5 (CH), 130.2 (C), 129.3 (CH), 126.1 (CH), 116.3 (C), 34.3 (CH_3_). HRMS (ESI^+^): Calcd for C_10_H_7_N_3_OS [M + H]^+^: 218.0388; Found: 218.03386.

*8-Isopropylthiazolo[5,4-f]quinazolin-9(8H)-one* (**8b**): Reaction time: 45 min; Yield: 99%; 145 mg; beige solid; R*_f_*: 0.69 (DCM/MeOH, 95:5; *v*/*v*); mp 237–240 °C; IR (neat) ν_max_ 3053, 2981, 2920, 1897, 1651, 1583, 1445, 1350, 1265, 1172, 827 cm^−1^; ^1^H-NMR (CDCl_3_, 25 °C, 300 MHz): *δ_H_* 9.19 (1H, s, H_2_), 8.44 (1H, d, *J* = 8.8 Hz, H_4_), 8.25 (1H, s, H_7_), 7.84 (1H, d, *J* = 8.8 Hz, H_5_), 5.37–5.12 (1H, m, NCH), 1.55 (3H, s, CH_3_), 1.53 (3H, s, CH_3_); ^13^C{^1^H}-NMR (CDCl_3_, 25 °C, 75.4 MHz): *δ_C_* 159.6 (C), 157.7 (CH), 152.5 (C), 146.6 (C), 143.4 (CH), 130.6 (C), 129.4 (CH), 126.0 (CH), 116.4 (C), 47.0 (CH), 22.2(2 × CH_3_). HRMS (ESI^+^): Calcd for C_12_H_11_N_3_OS [M + H]^+^: 246.0701; Found: 246.0708.

*8-Cyclopropylthiazolo[5,4-f]quinazolin-9(8H)-one* (**8c**), was described in ref [15], typical data: ^1^H-NMR (CDCl_3_, 300 MHz): *δ* = 9.22 (s, 1H, H2), 8.49 (d, *J* = 8.7 Hz, 1H, H4), 8.26 (s, 1H, H7), 7.87 (d, *J* = 8.7 Hz, 1H, H5), 3.50–3.25 (m, 1H, NCH), 1.37–1.17 (m, 2H, CH), 1.12–0.91 (m, 2H, CH). HRMS (ESI^+^): *m*/*z* calcd for C_12_H_10_N_3_OS: 244.0545; found: 244.0542.

*8-Cyclobutylthiazolo[5,4-f]quinazolin-9(8H)-one* (**8d**): Reaction time: 45 min; Yield: 97%; 148 mg; white solid; R*_f_*: 0.76 (DCM/MeOH, 95:5; *v*/*v*); mp 242–245 °C; IR (neat) ν_max_ 3045, 2992, 2957, 2879, 1655, 1601, 1585, 1349, 1277, 1163, 856 cm^−1^; ^1^H-NMR (CDCl_3_, 25 °C, 300 MHz): *δ_H_* 9.20 (1H, s, H_2_), 8.46 (1H, d, *J* = 8.8 Hz, H_4_), 8.31 (1H, s, H_7_), 7.86 (1H, d, *J* = 8.8 Hz, H_5_), 5.21–5.06 (1H, m, NCH), 2.71–2.55 (2H, m, CH_2_), 2.54–2.35 (2H, m, CH_2_), 2.05–1.90 (2H, m, CH_2_); ^13^C-NMR (CDCl_3_, 25 °C, 75.4 MHz): *δ_C_* 159.0 (C), 157.8 (CH), 152.6 (C), 146.8 (C), 143.8 (CH), 130.5 (C), 129.5 (CH), 126.1 (CH), 116.3 (C), 50.9 (CH), 29.9 (2 × CH_2_), 15.5 (CH_2_). HRMS (ESI^+^): Calcd for C_13_H_11_N_3_OS [M + H]^+^: 258.0701; Found: 258.0701.

*8-Cyclopentylthiazolo[5,4-f]quinazolin-9(8H)-one* (**8e**): Reaction time: 45 min; Yield: 98%; 160 mg; white solid; R*_f_*: 0.69 (DCM/MeOH, 95:5; *v*/*v*); mp 214–217 °C; IR (neat) ν_max_ 2960, 2874, 1661, 1585, 1348, 1259, 1154, 841, 818 cm^−1^; ^1^H-NMR (CDCl_3_, 25 °C, 300 MHz): *δ_H_* 9.22 (1H, s, H_2_), 8.49 (1H, d, *J* = 8.8 Hz, H_4_), 8.27 (1H, s, H_7_), 7.87 (1H, d, *J* = 8.8 Hz, H_5_), 5.36–5.20 (1H, m, NCH), 2.38–2.21 (2H, m, CH_2_), 2.12–1.74 (2H, m, CH_2_); ^13^C{^1^H}-NMR (CDCl_3_, 25 °C, 75.4 MHz): *δ_C_* 159.8 (C), 157.7 (CH), 152.3 (C), 146.4 (C), 144.1 (CH), 130.4 (C), 129.2 (CH), 125.8 (CH), 116.1 (C), 56.4 (CH), 32.1 (2 × CH_2_), 24.5 (2 × CH_2_). HRMS (ESI^+^): Calcd for C_14_H_13_N_3_OS [M + H]^+^: 272.0858; Found: 272.0855.

*8-Cyclohexylthiazolo[5,4-f]quinazolin-9(8H)-one* (**8f**): Reaction time: 90 min; Yield: 98%; 168 mg; white solid; R*_f_*: 0.75 (DCM/MeOH, 95:5; *v*/*v*); mp 227–230 °C; IR (neat) ν_max_ 3082, 3053, 2932, 2850, 1895, 1665, 1588, 1466, 1448, 1273, 1135, 845, 814 cm^−1^; ^1^H-NMR (CDCl_3_, 25 °C, 300 MHz): *δ_H_* 9.19 (1H, s, H_2_), 8.44 (1H, d, *J* = 8.8 Hz, H_4_), 8.25 (1H, s, H_7_), 7.83 (1H, d, *J* = 8.8 Hz, H_5_), 5.03–4.71 (1H, m, NCH), 2.20–1.87 (4H, m, CH_2_), 1.84–1.62 (3H, m, CH_2_), 1.62–1.41 (2H, m, CH_2_), 1.36–1.17 (1H, m, CH_2_); ^13^C{^1^H}-NMR (CDCl_3_, 25 °C, 75.4 MHz): *δ_C_* 159.6 (C), 157.7 (CH), 152.5 (C), 146.5 (C), 143.7 (CH), 129.4 (CH), 126.0 (CH), 116.4 (C), 54.3 (CH), 32.7 (2 × CH_2_), 26.0 (2 × CH_2_), 25.3 (CH_2_). HRMS (ESI^+^): Calcd for C_15_H_15_N_3_OS [M + H]^+^: 286.114; Found: 286.1021.

#### 3.2.2. General Procedure for the Synthesis of 8-Alkyl-2-(pyridin-3-yl)thiazolo[5,4-*f*]quinazolin-9(8*H*)-one (**4a**–**f**) from 8-Alkyl-thiazolo[5,4-*f*]quinazolin-9(8*H*)-one (**8a**–**f**)

Thiazolo[5,4-*f*]quinazolin-9(8*H*)-one **8a**–**f** (0.341 mmol), copper iodide (0.065 g, 0.341 mmol, 1 equiv), and TBD (95 mg, 0.682 mmol, 2.0 equiv) in dry DMF (850 µL) were added to a 2 mL glass vial, which was sealed under argon atmosphere. The mixture was stirred under microwave irradiation at 120 °C for 10 min. Then, Pd(OAc)_2_ (7.6 mg, 0.034 mmol, 10 mol%) and 3-iodopyridine (0.140 g, 0.682 mmol, 2.0 equiv) were added to the mixture and purged with argon. The reaction was then stirred under microwave irradiation at 120 °C for 5 h. The resulting solution was diluted with dichloromethane, and washed three times with a 5% aqueous ammonia solution, then with water and brine. The organic layer was dried over Na_2_SO_4_ and concentrated under vacuum. The crude product was purified by flash chromatography on silica gel with MeOH/CH_2_Cl_2_ as eluent (1/0 to 95:5; *v*/*v*), to afford the corresponding product.

*8-Methyl-2-(pyridin-3-yl)thiazolo[5,4-f]quinazolin-9(8H)-one* (**4a**): Yield: 14%; 14 mg; Yellow solid; R*_f_*: 0.36 (DCM/MeOH, 95:5; *v*/*v*); mp 272–275 °C; IR (neat) ν_max_ 3068, 1651, 1588, 1446, 1346, 699 cm^−1^; ^1^H-NMR (CDCl_3_, 25 °C, 300 MHz): *δ_H_* 9.39 (1H, br s, H_Ar_), 8.73 (1H, d, *J* = 4.8 Hz, H_Ar_), 8.58–8.36 (2H, m, H_Ar_ + H_4_), 8.19 (1H, s, H_7_), 7.86 (1H, d, *J* = 8.8 Hz, H_5_), 7.47 (1H, dd, *J* = 8.0, 4.8 Hz, H_Ar_), 3.73 (3H, s, CH_3_); ^13^C{^1^H}-NMR (CDCl_3_, 25 °C, 75.4 MHz): *δ_C_* 168.36 (C), 160.40 (C), 153.29 (C), 151.82 (CH), 148.66 (CH), 147.20 (C), 146.60 (CH), 134.71 (CH), 131.54 (C), 129.77 (C), 129.25 (CH), 126.60 (CH), 124.06 (CH), 116.48 (C), 34.37 (CH_3_). HRMS (ESI^+^): Calcd for C_15_H_10_N_4_OS [M + H]^+^: 295.0654; Found: 295.0668.

*8-Isopropyl-2-(pyridin-3-yl)thiazolo[5,4-f]quinazolin-9(8H)-one* (**4b**): Yield: 64%; 70 mg; Yellow solid; R*_f_*: 0.46 (DCM/MeOH, 95:5; *v*/*v*); mp 202–205 °C; IR (neat) ν_max_ 3540, (C), 151.82 (CH), 148.66 (CH), 147.20 (C), 146.60 (CH), 134.71 (CH), 131.54 (C), 129.77 (C), 129.25 (CH), 126.60 (CH), 124.06 (CH), 116.48 (C), 34.37 (CH_3_). HRMS (ESI^+^): Calcd for C_15_H_10_N_4_OS [M + H]^+^: 295.0654; Found: 295.0668.

*8-Cyclopropyl-2-(pyridin-3-yl)thiazolo[5,4-f]quinazolin-9(8H)-one* (**4c** *or* **FC162**) [34]: yield: 69%; 91 mg; beige powder; R*_f_*: 0.45 (DCM/ EtOAc, 1/1, *v*/*v*); mp: 263–266°C; IR (neat) ν_max_ 3059, 3012, 2114, 1659, 1588, 1449, 1344, 1295, 1024, 837 cm^−1^; ^1^H-NMR (CDCl_3_, 25 °C, 300 MHz): *δ_H_* 9.41 (d, *J* = 2.3 Hz, 1H, H_Ar_), 8.75 (d, *J* = 4.9 Hz, 1H, H_Ar_), 8.54–8.38 (m, 2H, H_Ar_ + H_4_), 8.26 (s, 1H, H_7_), 7.88 (d, *J* = 8.7 Hz, 1H, H_5_), 7.48 (dd, *J* = 8.0, 4.9 Hz, 1H, CH_Ar_), 3.45–3.26 (m, 1H, NCH), 1.36–1.24 (m, 2H, CH), 1.14–0.96 (m, 2H, CH). ^13^C-NMR (CDCl_3_, 75.4 MHz): *δ* = 168.5 (C), 161.3 (C), 153.4 (C), 151.9 (CH), 148.7 (CH), 146.7 (CH), 146.6 (C), 134.7 (CH), 131.7 (C), 129.8 (C), 129.3 (CH), 126.6 (CH), 124.1 (CH), 116.4 (C), 29.8 (CH), 6.7(2 × CH_2_). HRMS (ESI^+^): *m*/*z* calcd for ^12^C_17_^1^H_12_^14^N_4_^16^O^32^S [M + H]^+^: 321.0807; Found: 321.0810.

*8-Cyclobutyl-2-(pyridin-3-yl)thiazolo[5,4-f]quinazolin-9(8H)-one* (**4d**): Yield: 57%; 64 mg; Yellow solid; R*_f_*: 0.45 (DCM/MeOH, 95:5; *v*/*v*); mp 251–254 °C; IR (neat) ν_max_ 3072, 2992, 2948, 2865, 1906, 1655, 1584, 1347, 848 cm^−1^; ^1^H-NMR (CDCl_3_ + D_2_O, 25 °C, 300 MHz): *δ_H_* 9.39 (1H, br s, H_Ar_), 8.72 (1H, d, *J* = 4.8, H_Ar_), 8.45–8.29 (2H, m, H_Ar_ + H_4_), 8.30 (1H, s, H_7_), 7.86 (1H, d, *J* = 8.8 Hz, H_5_), 7.46 (1H, dd, *J* = 8.0, 4.8 Hz, H_Ar_), 5.18–5.09 (1H, m, NCH), 2.70–2.60 (2H, m, CH_2_), 2.54–2.40 (2H, m, CH_2_), 2.04–1.94 (2H, m, CH_2_); ^13^C{^1^H}-NMR (CDCl_3_ + D_2_O, 25 °C, 75.4 MHz): *δ_C_* 168.2 (C), 160.0 (C), 153.2 (C), 151.8 (CH), 148.6 (CH), 146.7 (C), 143.8 (CH), 134.7 (CH), 131.6 (C), 129.8 (C), 129.2 (CH), 126.5 (CH), 124.1 (CH), 116.3 (C), 51.1 (CH), 29.8 (2 × CH_2_), 15.5 (CH_2_). HRMS (ESI^+^): Calcd for C_18_H_14_N_4_OS [M + H]^+^: 335.0967; Found: 335.0974.

*8-Cyclopentyl-2-(pyridin-3-yl)thiazolo[5,4-f]quinazolin-9(8H)-one* (**4e**): Yield: 65%; 77 mg; Yellow solid; R*_f_*: 0.43 (DCM/MeOH, 95:5; *v*/*v*); mp 212–215 °C; IR (neat) ν_max_ 3064, 3964, 2872; 1902, 1645, 1584, 1451, 828 cm^−1^; ^1^H-NMR (CDCl_3_, 25 °C, 300 MHz): *δ_H_* 9.40 (1H, br s, H_Ar_), 8.73 (1H, d, *J* = 4.8 Hz, H_Ar_), 8.50–8.38 (2H, m, H_Ar_ + H_4_), 8.25 (1H, s, H_7_), 7.86 (1H, d, *J* = 8.8 Hz, H_5_), 7.46 (dd, *J* = 8.0, 4.8 Hz, H_Ar_), 5.39–5.08 (1H, m, NCH), 2.46–2.16 (2H, m, CH_2_), 2.14–1.67 (4H, m, CH_2_); ^13^C{^1^H}-NMR (CDCl_3_, 25 °C, 75.4 MHz): *δ_C_* 168.0 (C), 159.8 (C), 152.9 (C), 151.5 (CH), 148.4 (CH), 146.3, 144.1 (CH), 134.4 (CH), 131.5 (C), 129.8 (C), 128.9 (CH), 126.2 (CH), 124.1 (CH), 116.2 (C), 56.8 (CH), 32.1 (2 × CH_2_), 24.6 (2 × CH_2_). HRMS (ESI^+^): Calcd for C_19_H_16_N_4_OS [M + H]^+^: 349.1123; Found: 349.1115.

*8-Cyclohexyl-2-(pyridin-3-yl)thiazolo[5,4-f]quinazolin-9(8H)-one* (**4f**): Yield: 54%; 67 mg; Yellow solid; R*_f_*: 0.43 (DCM/MeOH, 95:5; *v*/*v*); mp 201–204 °C; IR (neat) ν_max_ 3309, 3146, 1076, 2920, 1902, 1661, 1587, 1350, 1333, 826 cm^−1^; ^1^H-NMR (CDCl_3_, 25 °C, 300 MHz): *δ_H_* 9.38 (1H, br s, H_Ar_), 8.72 (1H, br s, H_Ar_), 8.56–8.35 (2H, m, H_Ar_ + H_4_), 8.25 (1H, s, H_7_), 7.84 (1H, d, *J* = 8.7 Hz, H_4_), 7.45 (1H, dd, *J* = 8.0, 4.8 Hz, H_Ar_), 4.96–4.79 (1H, m, NCH), 2.16–1.89 (4H, m, CH_2_), 1.86–1.65 (3H, m, CH_2_), 1.65–1.43 (2H, m, CH_2_), 1.38–1.16 (1H, m, CH_2_); ^13^C{^1^H}-NMR (CDCl_3_, 25 °C, 75.4 MHz): *δ_C_* 168.1 (C), 159.6 (C), 153.0 (C), 151.6 (CH), 148.5 (CH), 146.3 (C), 143.7 (CH), 134.5 (CH), 131.7 (C), 129.8 (C), 129.1 (CH), 126.3 (CH), 124.0 (CH), 116.3 (C), 54.5 (CH), 32.6 (2 × CH_2_), 25.9 (2 × CH_2_), 25.2 (CH_2_). HRMS (ESI^+^): Calcd for C2_0_H_18_N_4_OS [M + H]^+^: 363.1280; Found: 363.1295.

### 3.3. In Vitro Kinase Preparation and Assays

#### 3.3.1. Buffers

Homogenization buffer: 25 mM MOPS; 15 mM EGTA; 15 mM MgCl_2_; 60 mM β-glycerophosphate; 15 mM *p*-nitrophenylphosphate; 2 mM dithiothreitol (DTT); 1 mM Na_3_VO_4_; 1 mM NaF; 1 mM di-sodium phenylphosphate; 1× protease inhibitor cocktail; 0.2% Nonidet P-40 substitute.

Buffer A: 10 mM MgCl_2_; 1 mM EGTA; 1 mM DTT; 25 mM Tris/HCl, and 50 μg/mL heparin.

Buffer B: 60 mM β-glycerophosphate; 30 mM *p*-nitrophenylphosphate; 25 mM MOPS pH 7.0; 5 mM EGTA; 15 mM MgCl_2_; 1 mM DTT; and 0.1 mM sodium vanadate.

All chemicals were purchased from Sigma-Aldrich (St. Quentin Fallavier, France), unless otherwise stated, and the protease inhibitor cocktail was from Roche (Boulogne-Billancourt, France).

#### 3.3.2. Kinase Preparations and Assays

Kinase activities were assayed in triplicates, in buffer A or B, at 30 °C, at a final adenosine triphosphate (ATP) concentration of 15 μmol/L. Blank values were subtracted, and activities were expressed in percent (%) of the maximal activity, i.e., in the absence of inhibitors. Controls were performed with appropriate dilutions of dimethyl sulfoxide (DMSO).

*CDK5/p25* (Human, recombinant) was prepared as previously described [37]. Its kinase activity was assayed in buffer A, with 1 mg of histone H1/mL, in the presence of 15 μM [γ-^33^P] ATP (3000 Ci/mmol; 10 mCi/mL) in a final volume of 30 μL. After 30 min incubation at 30 °C, 25 μL aliquots of supernatant were spotted onto sheets of Whatman P81 phosphocellulose paper, and 20 s later, the filters were washed eight times (for at least 5 min each time) in a solution of 10 mL phosphoric acid/L of water. The wet filters were counted in the presence of 1 mL ACS (Amersham) scintillation fluid.

*GSK-3α*/*β* (porcine brain, native) was assayed, in buffer A, with 0.5 mg BSA/mL + 1 mM DTT, using GS-1 (YRRAAVPPSPSLSRHSSPHQSpEDEEE) (pS stands for phosphorylated serine), a GSK-3 specific substrate [38], in the presence of 15 μmol/L [γ-^33^P] ATP (3000 Ci/mmol; 10 mCi/mL) in a final volume of 30 μL. After 30 min incubation at 30 °C, the reaction was stopped by harvesting onto P81 phosphocellulose supernatant (Whatman, Dutscher SAS, Brumath, France) using a FilterMate harvester (PerkinElmer, Courtaboeuf, France), and were washed in 1% phosphoric acid. Scintillation fluid was added, and the radioactivity measured in a Packard counter.

*CLK1* (Human, recombinant, expressed in *E. coli* as GST fusion protein) was assayed in buffer A (+0.15 mg BSA/mL) with RS peptide (GRSRSRSRSRSR) (1 μg/assay).

*CK1δ*/*ε* (porcine brain, native) was assayed as described for CDK1, but in buffer B, and using 25 μM CKS peptide (RRKHAAIGpSAYSITA), a CK1-specific substrate [39].

*DYRK1A* (Human, recombinant, expressed in *E. coli* as GST fusion proteins) was purified by affinity chromatography on glutathione-agarose and assayed as described for CDK1/cyclin B, with with 0.5 mg BSA/mL + 1 mM DTT and using Woodtide (KKISGRLSPIMTEQ) (1.5 μg/assay) as a substrate, a residue of transcription factor FKHR.

## 4. Conclusions

This work demonstrates the efficacy of synthetic methodologies, such as C–H arylation of arenes and hetero-arenes for SAR studies. The application of this powerful tool at the last stage of the synthesis of kinase inhibitors allowed the synthesis of arrays of molecules inspired by fragment-growing studies generated by molecular modeling calculations. Among the potential active compounds generated through this strategy, **FC162** (**4c**) was found to be the best candidate for development as a DYRK inhibitor.

## Figures and Tables

**Table 1:** Chemical structures and yields obtained for the synthesis ^a^ of series **2a**–**j** (R^1^, R^2^, and R^3^) and **3a**–**c** ^b^ (R, X, and Y).

**Product**	**R^1^**	**R^2^**	**R^3^**	**X(Ar)**	**Yield ^c^ (%)**
**2a**	H	H	H	Br	92
**2b**	Me	H	H	Br	86
**2c**	MeO	H	H	I	63
**2d**	Cl	H	H	I	87
**2e**	F	H	H	I	71
**2f**	CN	H	H	I	59
**2h**	NMe_2_	H	H	Br	87
**2i**	Cl	H	Cl	I	62
**2j**	Cl	Cl	H	I	64
**Product**	**X**	**Y**	**R**	**X(Ar)**	**Yield ^c^ (%)**
**3a**	CH	N	H	I	69
**3b**	N	N	H	I	47
**3c**	CH	N	OMe	I	29

^a^ Premixing **1**, DBU, and CuI, 10 min before adding ArI or ArBr, Pd(OAc)_2_, and stirring for 5 h; ^b^ In the case of 3-iodopyridine, TBD [31] was used as base instead of DBU; ^c^ Isolated yields.

**Table 2:** Chemical structures (R^1^) and yields obtained for the synthesis of series **7a**–**f**, **8a**–**f**, and **4a**–**f**.

–R^1^	Compound	Yield ^a^ (%)	Compound	Yield ^a^ (%)	Compound	Yield ^a^ (%)
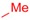	**7a**	70	**8a**	98	**4a**	14
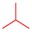	**7b**	43	**8b**	99	**4b**	64
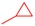	**7c**	86	**8c**	86	**4c**	43
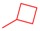	**7d**	65	**8d**	97	**4d**	57
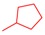	**7e**	60	**8e**	98	**4e**	55
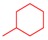	**7f**	50	**8f**	98	**4f**	54

^a^ Isolated yield.

**Table 3:** Chemical structures and yields obtained for the synthesis ^a^ of series **9a**–**j** (R^1^, R^2^, and R^3^).

Product	R^1^	R^2^	R^3^	X(Ar)	Yield ^b^ (%)
**9a**	H	H	H	Br	58
**9b**	Me	H	H	Br	64
**9c**	MeO	H	H	I	76
**9d**	Cl	H	H	I	67
**9e**	F	H	H	I	52
**9f**	CN	H	H	I	- ^c^
**9h**	NMe_2_	H	H	Br	31
**9i**	Cl	H	Cl	I	65
**9j**	Cl	Cl	H	I	69

^a^ Premixing **1**, DBU, and CuI, 10 min before adding ArI or ArBr, Pd(OAc)_2_ and stirring for 5 h; ^b^ Isolated yields; ^c^ Not prepared.

**Table 4:** Kinase inhibitory activity ^a,b,c^ of the thiazolo[5,4-*f*]quinazolin- series (**2a**–**j**, **3a**–**c**, **4a**–**i**, and **10**).

Compounds	CDK5/p25	CK1δ/ε	CLK1	DYRK1A	GSK-3α/β
**2a–j**	>10	>10	>10	>10	>10
**3a**	>10	>10	2.0	0.012	3.7
**3b**	>10	>10	3.33	0.133	6.0
**3c**	>10	>10	>10	>10	>10
**4a**	n.t. ^d^	>10	>10	>10	>10
**4b**	n.t.	>10	>10	>10	>10
**4c (FC162)**	n.t.	6.0	0.018	0.011	0.068
**4d**	n.t.	>10	>10	>10	>10
**4e**	n.t.	>10	>10	>10	>10
**4f**	n.t.	>10	>10	>10	>10
**9a–i**	>10	>10	>10	>10	≥10
**10**	>10	>10	>10	>10	≥10
Harmine	>10	1.5	0.026	0.029	>10

^a^ IC_50_ values are reported in μM.; ^b^ Kinase activities were assayed in triplicate; ^c^ Typically, the standard deviation of single data points was below 10%; ^d^ n.t., not tested.

## Supplementary Materials

The following are available online. ^1^H-NMR and ^13^C-NMR spectra of new compounds **8a**–**f** and **4a**–**f**.

## Abbreviations

ATP	adenosine triphosphate
CMGC group	group of kinases including cyclin-dependent kinases (CDKs), mitogen-activated protein kinases (MAP kinases), glycogen synthase kinases (GSK) and Cdc2-like kinases (CLKs)
DBU	1,8-diazabicyclo[5.4.0]undec-7-ene
DMF	*N*,*N*-dimethylformamide
DMFDMA	*N*,*N*-dimethylformamide dimethyl acetal
NBS	*N*-bromosuccinimide
TBD	1,5,7-triazabicyclo[4.4.0]dec-5-ene
SAR	structure–activity relationship
