# Supplementary material for: Development of Kinase Inhibitors via Metal-Catalyzed C–H Arylation of 8-Alkyl-thiazolo[5,4-f]-quinazolin-9-ones Designed by Fragment-Growing Studies
Source: Molecules. 2018 Aug 29;23(9):2181. doi: 10.3390/molecules23092181 (PMC6225322; doi:10.3390/molecules23092181)

molecules

Article

Development of kinase inhibitors via metal-catalyzed C-H arylation of 8-alkyl-thiazolo[5,4-*f*]-quinazolin-9-ones designed by fragment-growing studies.

Florence Couly [1], Marine Harari [1], Carole Dubouilh-Benard [1], Laetitia Bailly [1], Emilie Petit [1], Julien Diharce [2], Pascal Bonnet [2], Laurent Meijer [3], Corinne Fruit [1,]* and Thierry Besson [1,]*

[1] Normandie Univ, UNIROUEN, INSA Rouen, CNRS, COBRA UMR 6014, 76000 Rouen, France.; florence.couly@insa-rouen.fr; marine.harari@etu.univ-rouen.fr; carole.dubouilh@univ-rouen.fr; laetitia.bailly@insa-rouen.fr; emilie.petit@insa-rouen.fr
[2] Institut de Chimie Organique et Analytique (ICOA), Université d'Orléans, UMR CNRS, 7311 BP 6759, 45067 Orléans Cedex 2, France; julien.diharce@univ-orleans.fr; pascal.bonnet@univ-orleans.fr
[3] ManRos Therapeutics, Perharidy Peninsula, 29680-Roscoff, France; lmeijer@manros-therapeutics.com
* Correspondence: corinne.fruit@univ-rouen.fr; thierry.besson@univ-rouen.fr; Tel.: +33-235-522-904

Supporting information

Compounds **1** and **7a-f** were described in ref [15].

Compounds **2a-i** and **3a-b** were described in ref [31]

Compounds **2j**, **3c**, **4c**, **9-j** and **10** were described in ref [32];
The new products **8a-f** and **4a**, **4b** and **4d-f** are described below. The lead molecule FC162 (**4c**) is described again.

^{1}H NMR and ^{13}C NMR spectra of new compounds **8a-f** and **4a-f**. pp 2-13

8-Methylthiazolo[5,4-*f*]quinazolin-9(8*H*)-one (**8a**)

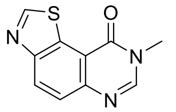

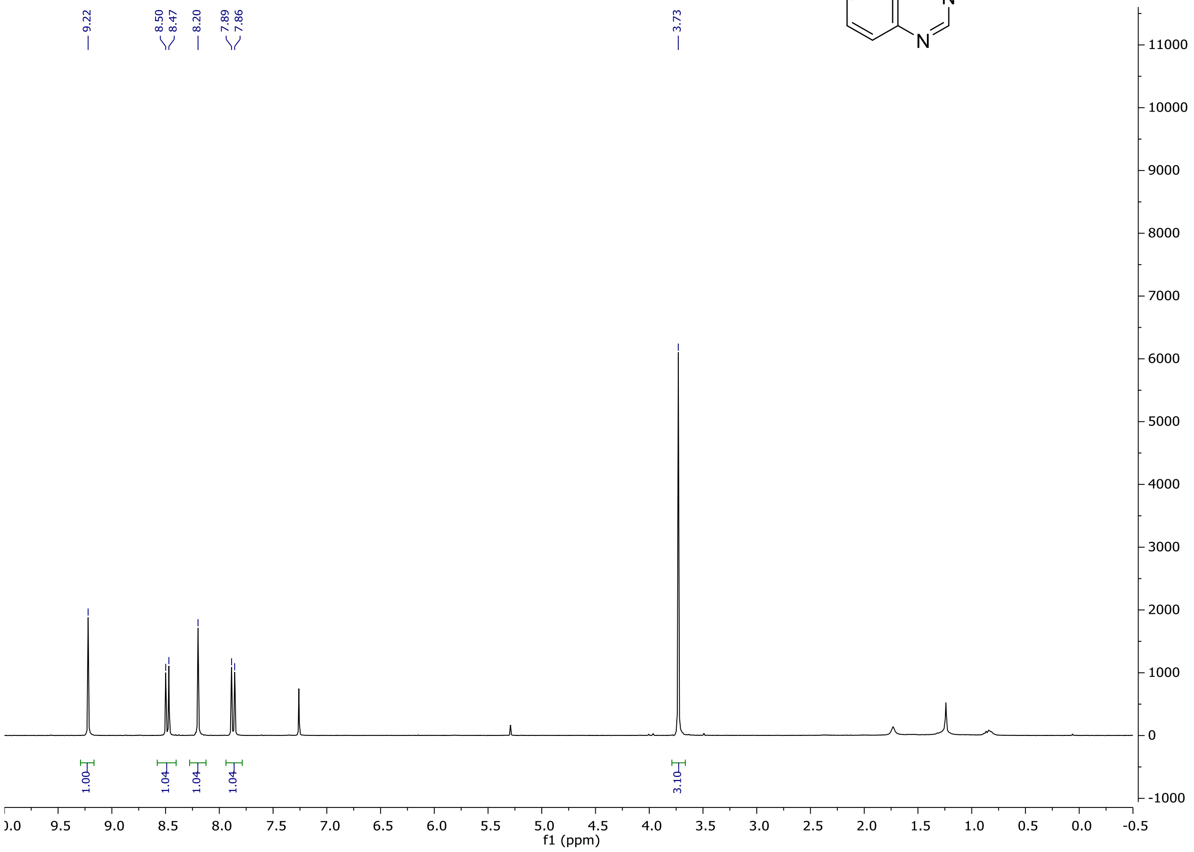

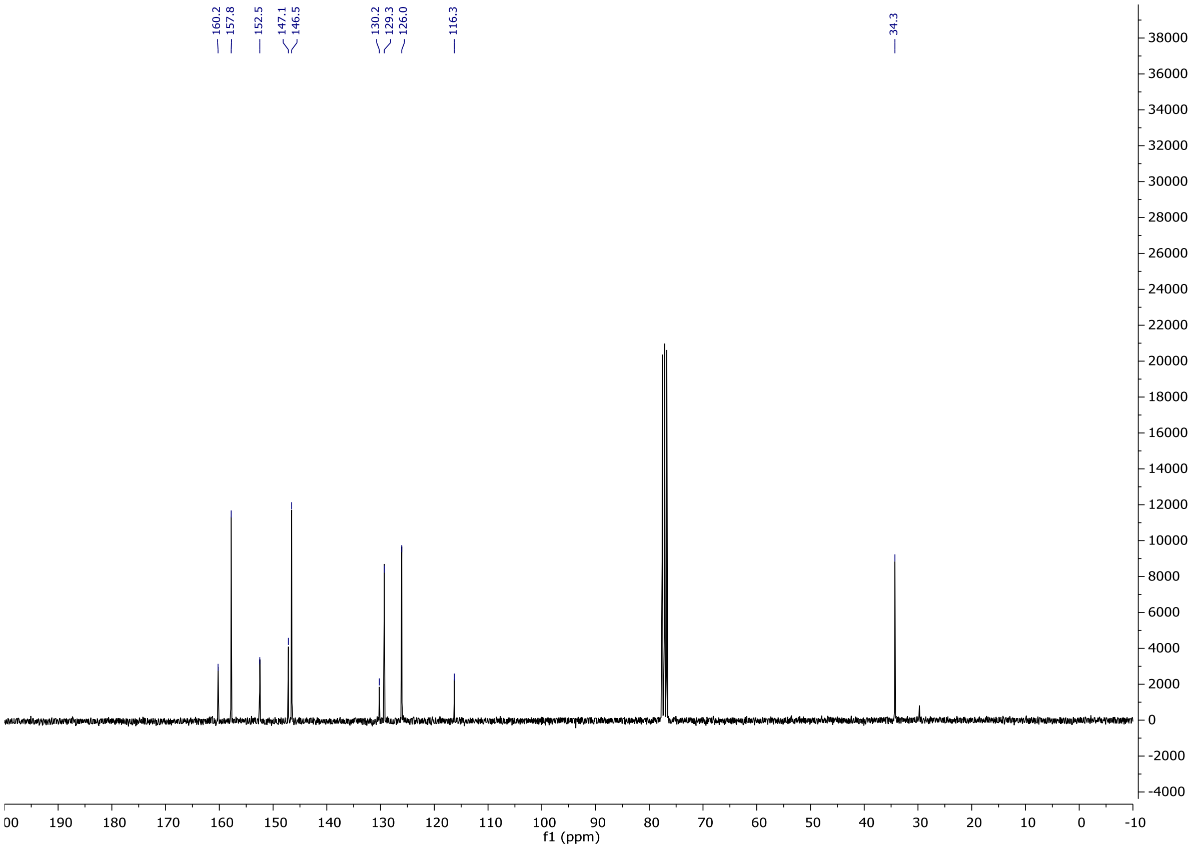

8-Isopropylthiazolo[5,4-*f*]quinazolin-9(8*H*)-one (**8b**)

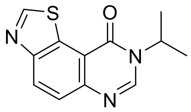

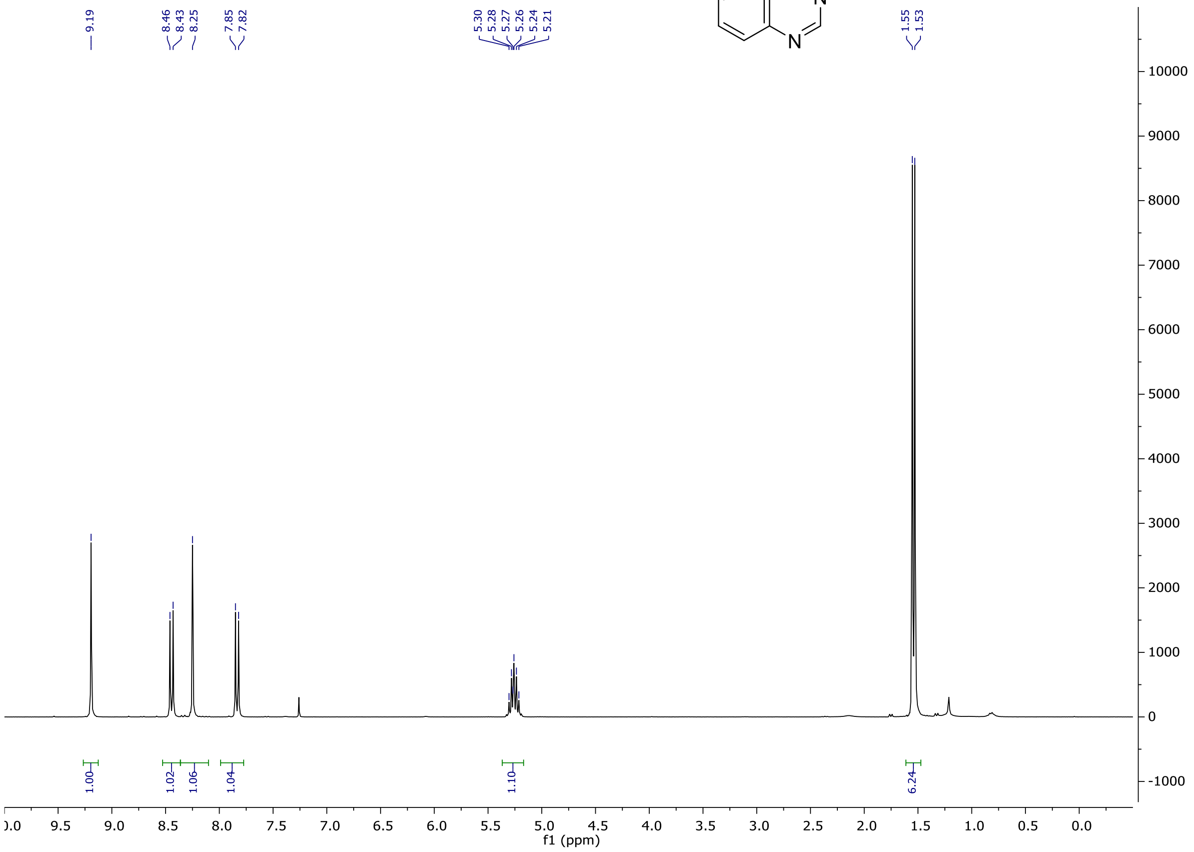

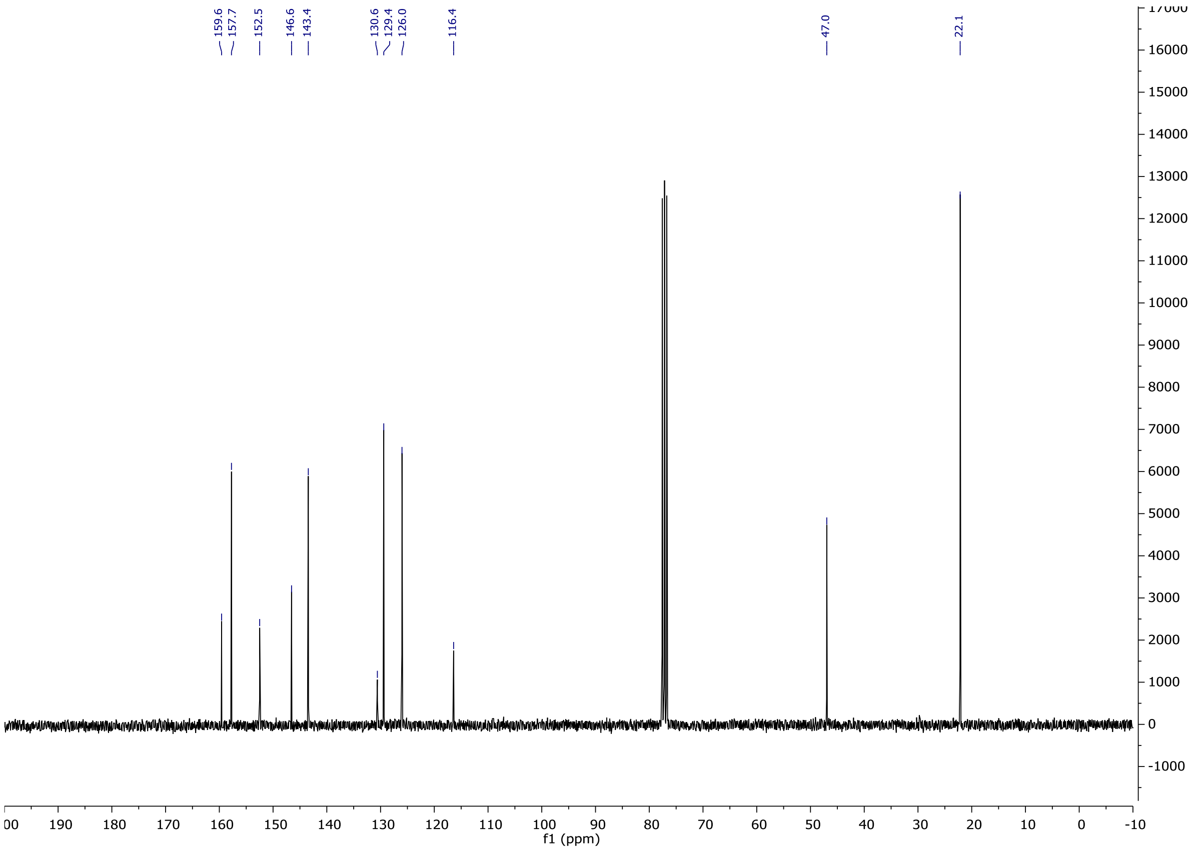

8-Cyclopropylthiazolo[5,4-*f*]quinazolin-9(8*H*)-one (**8c**)

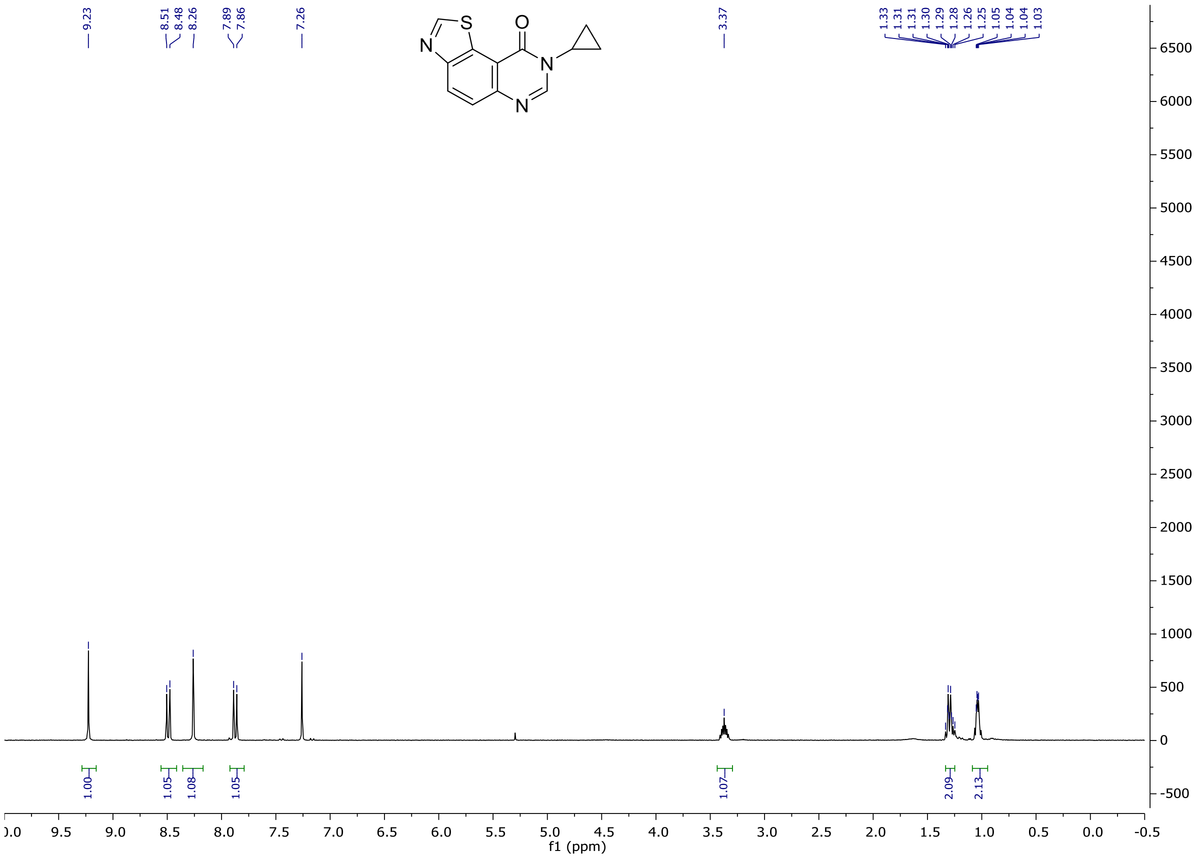

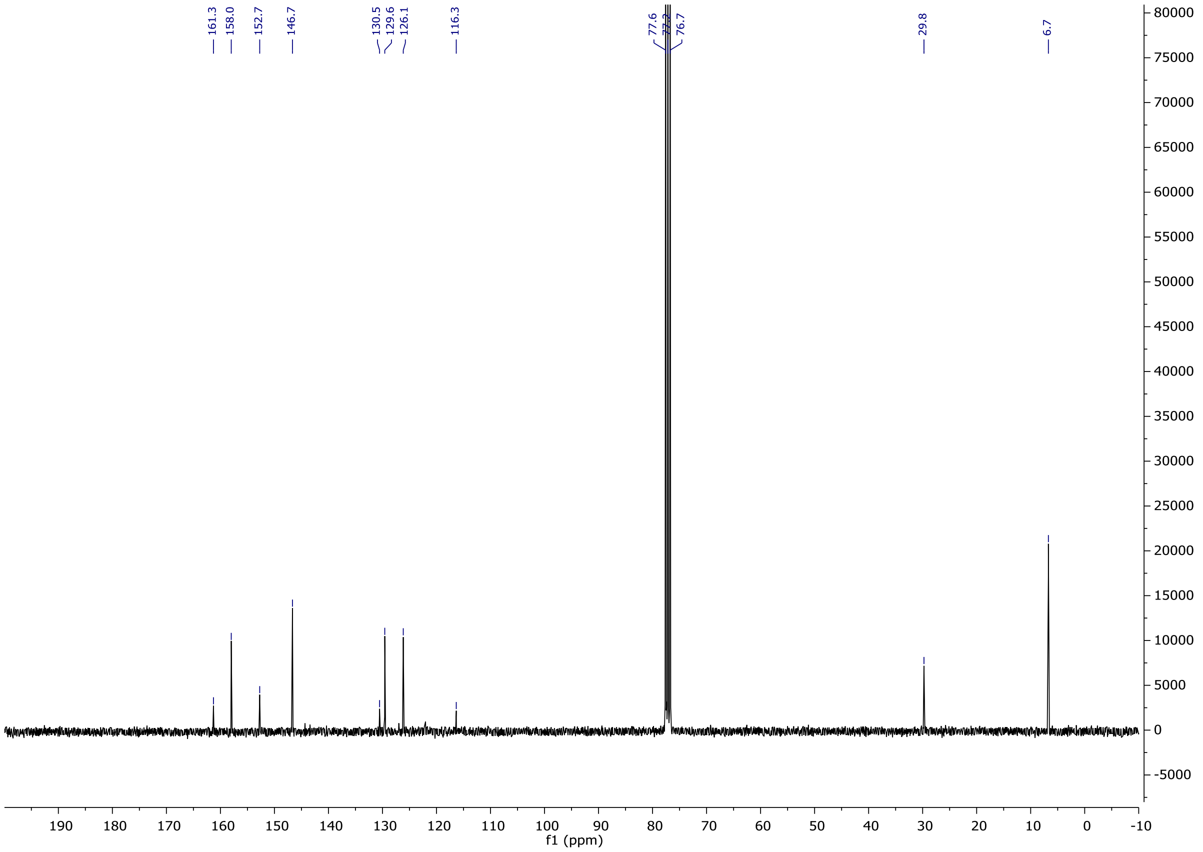

8-Cyclobutylthiazolo[5,4-*f*]quinazolin-9(8*H*)-one (**8d**)

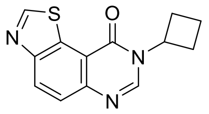

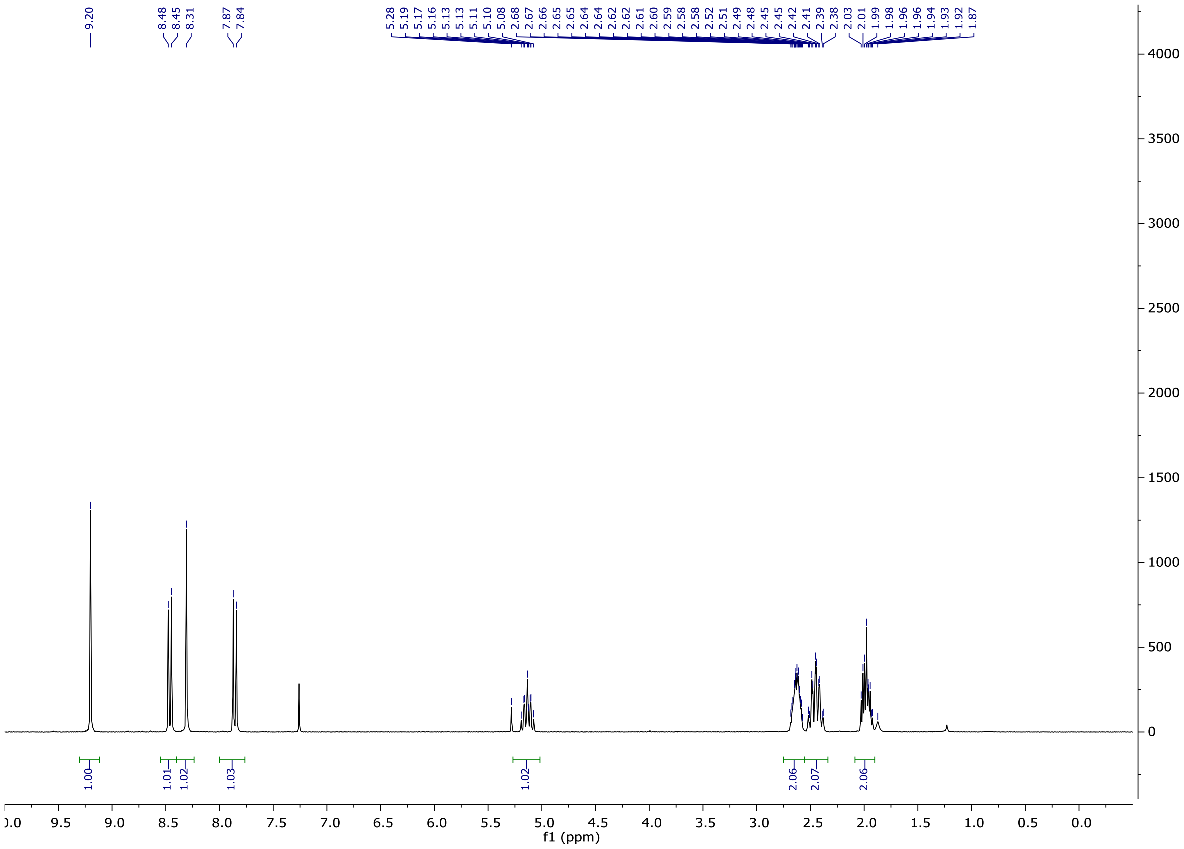

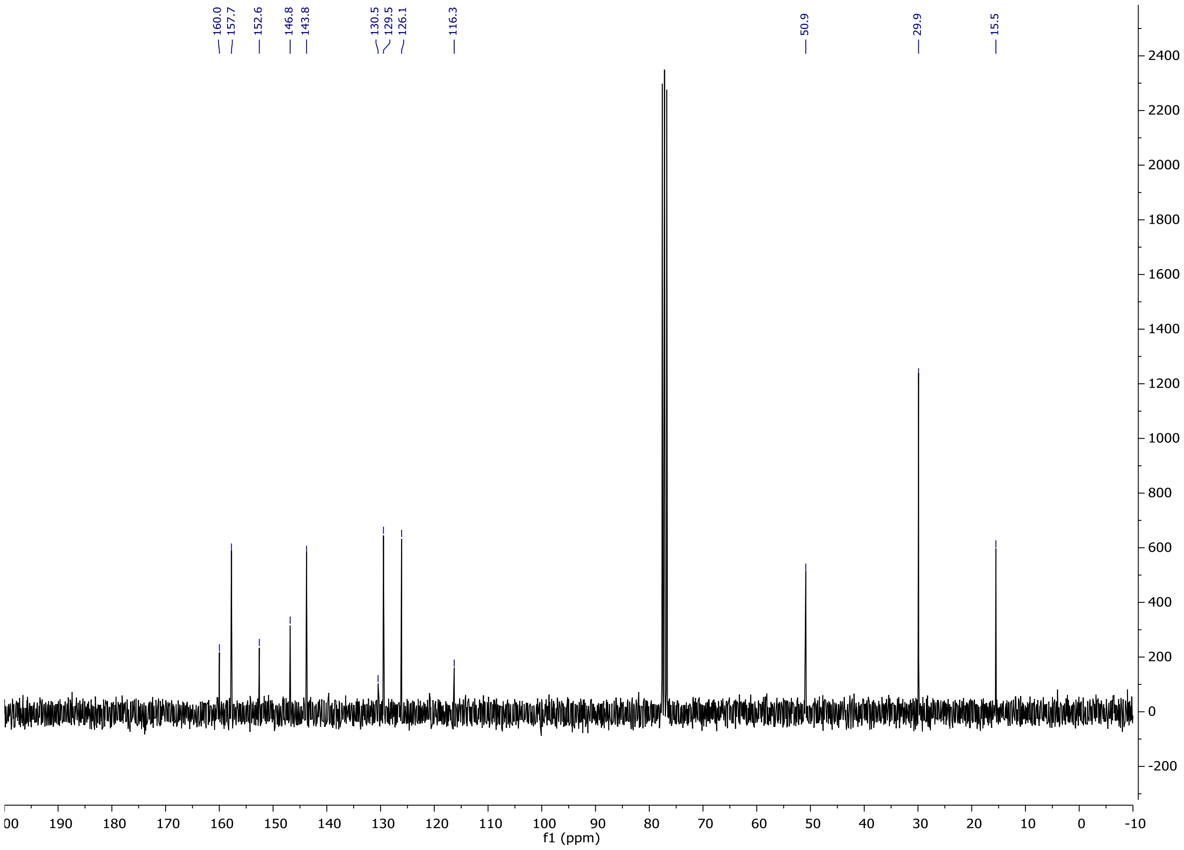

8-Cyclopentylthiazolo[5,4-*f*]quinazolin-9(8*H*)-one (**8e**)

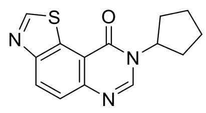

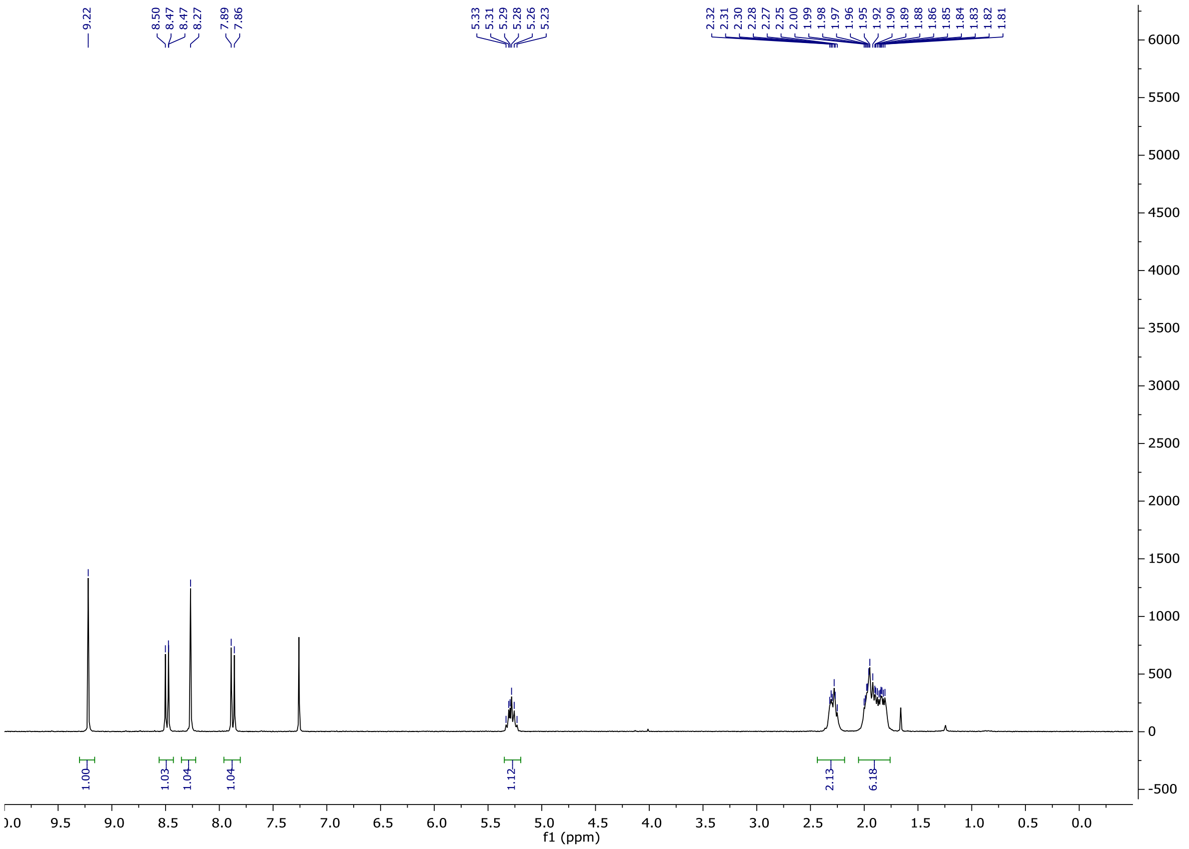

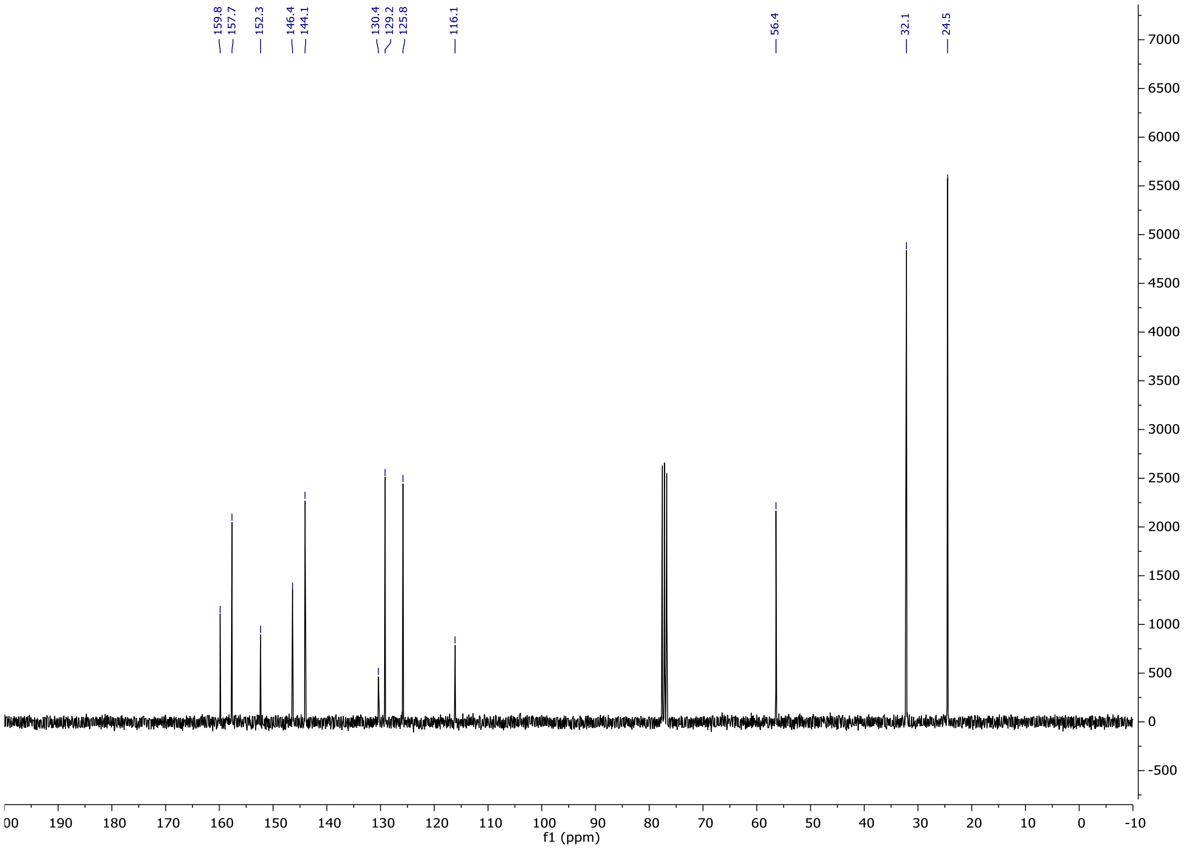

8-Cyclohexylthiazolo[5,4-*f*]quinazolin-9(8*H*)-one (**8f**)

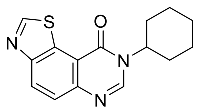

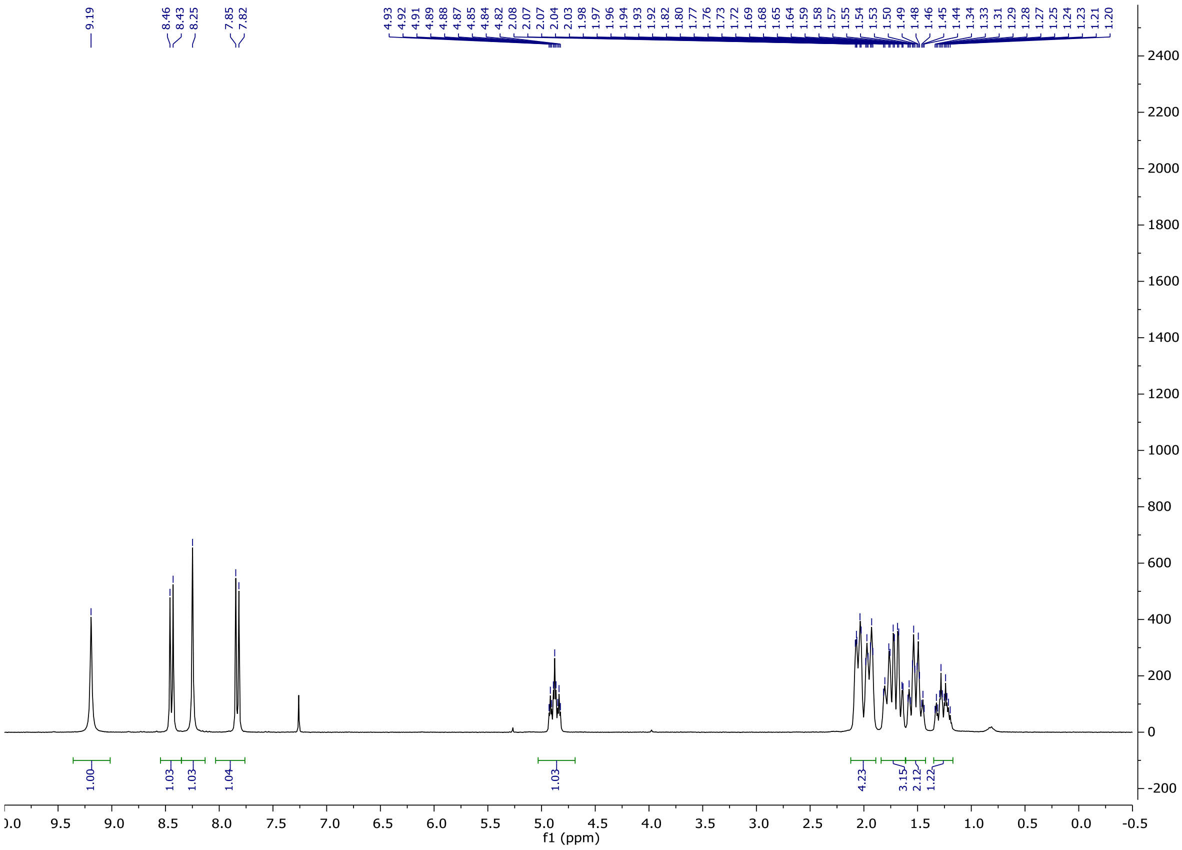

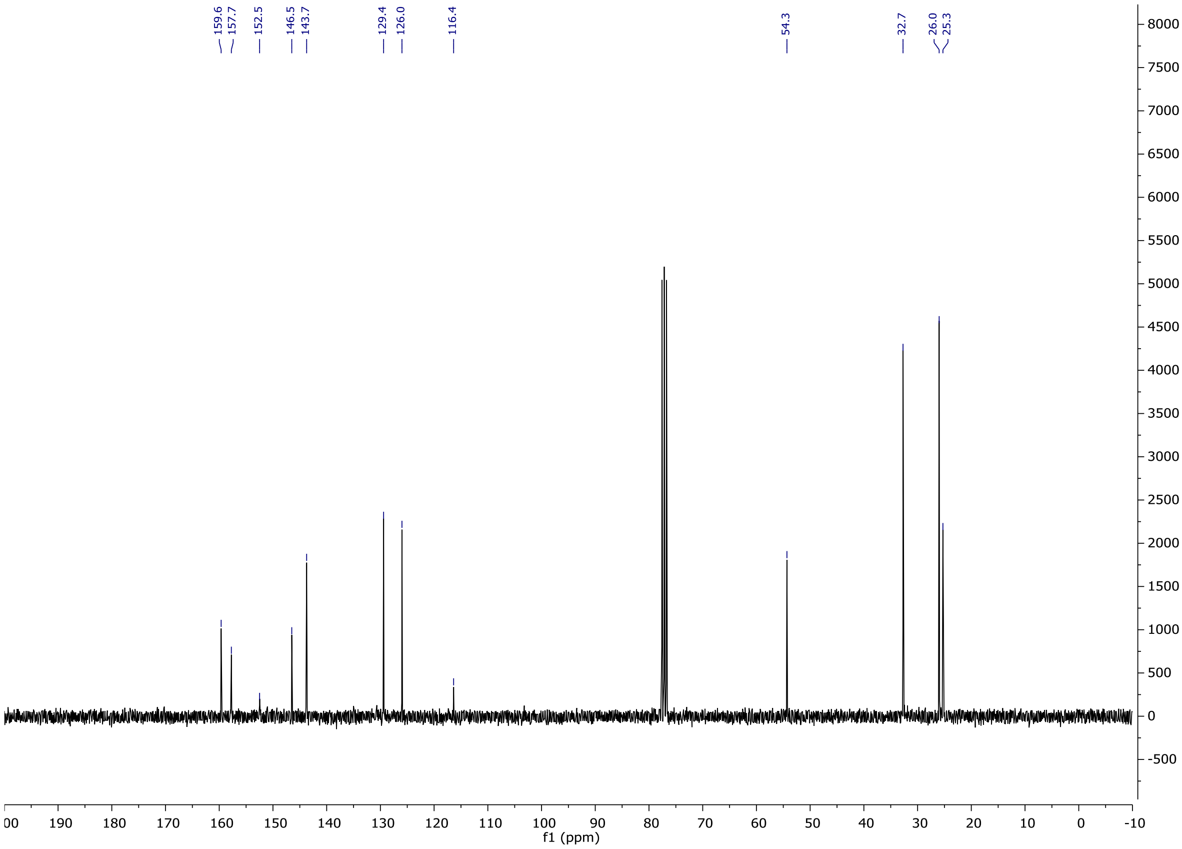

8-Methyl-2-(pyridin-3-yl)thiazolo[5,4-*f*]quinazolin-9(8*H*)-one (**4a**)

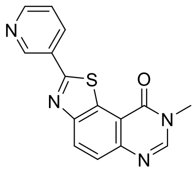

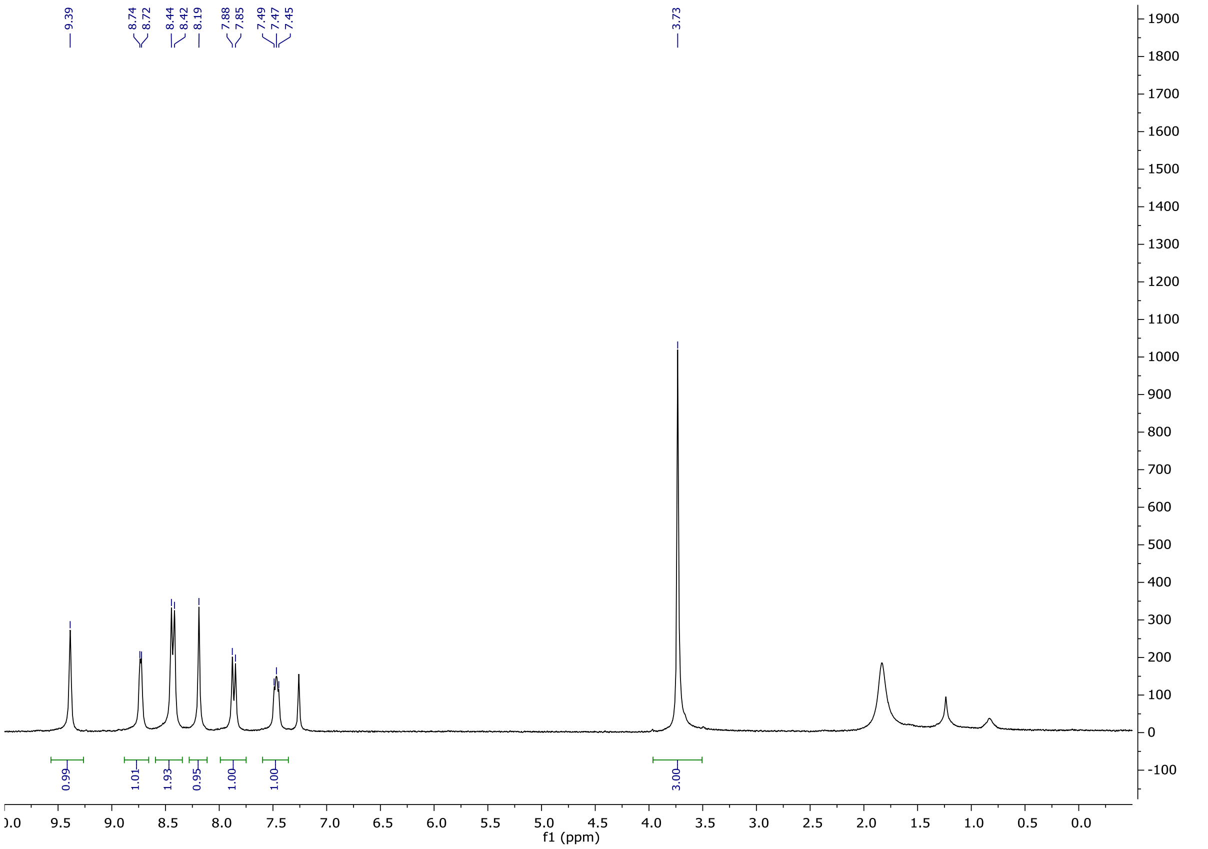

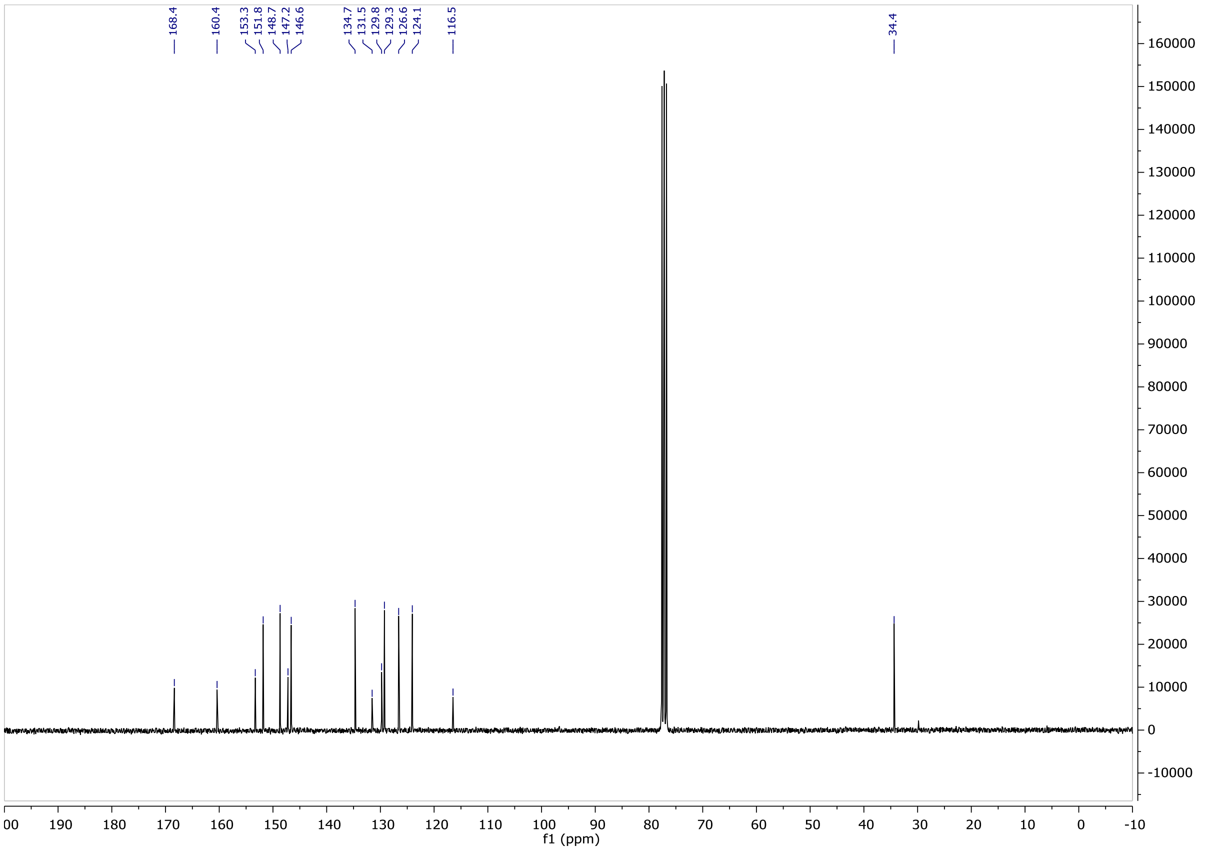

8-Isopropyl-2-(pyridin-3-yl)thiazolo[5,4-*f*]quinazolin-9(8*H*)-one (**4b**)

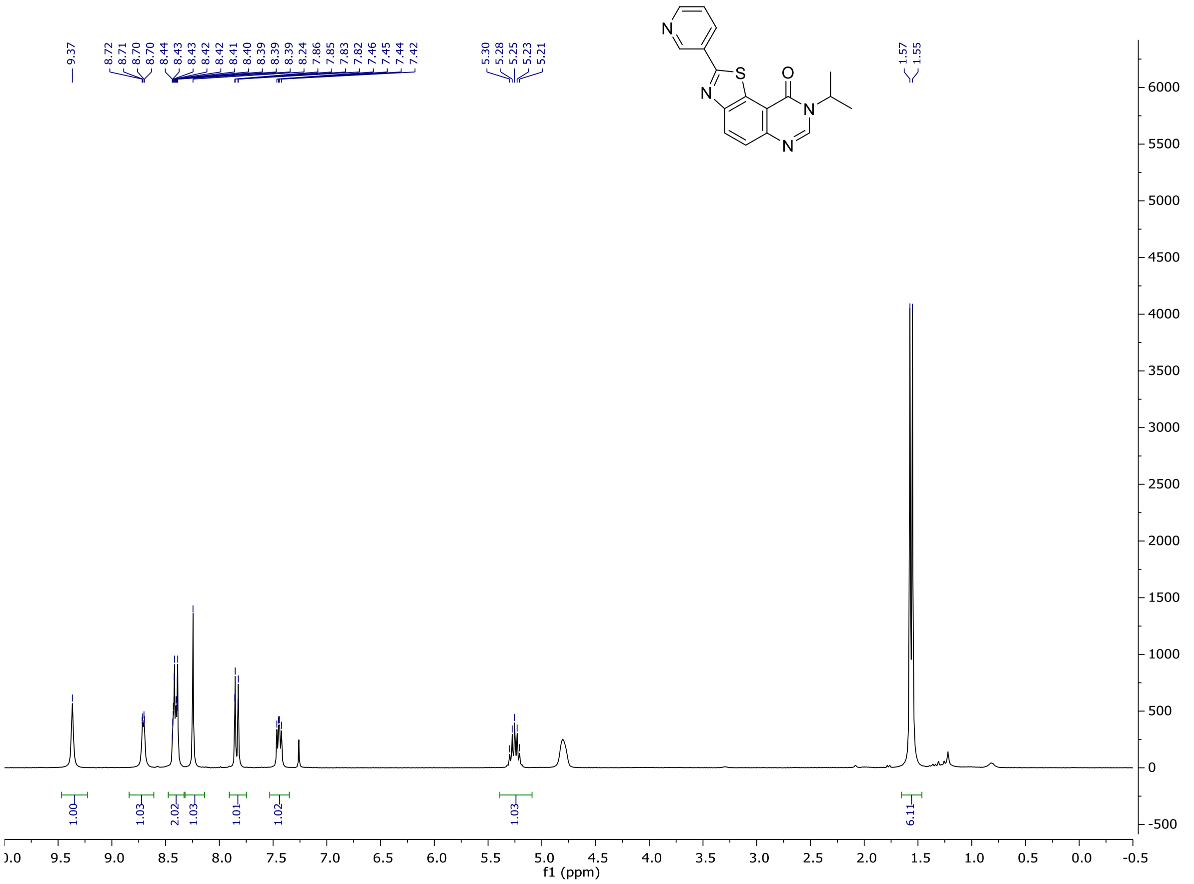

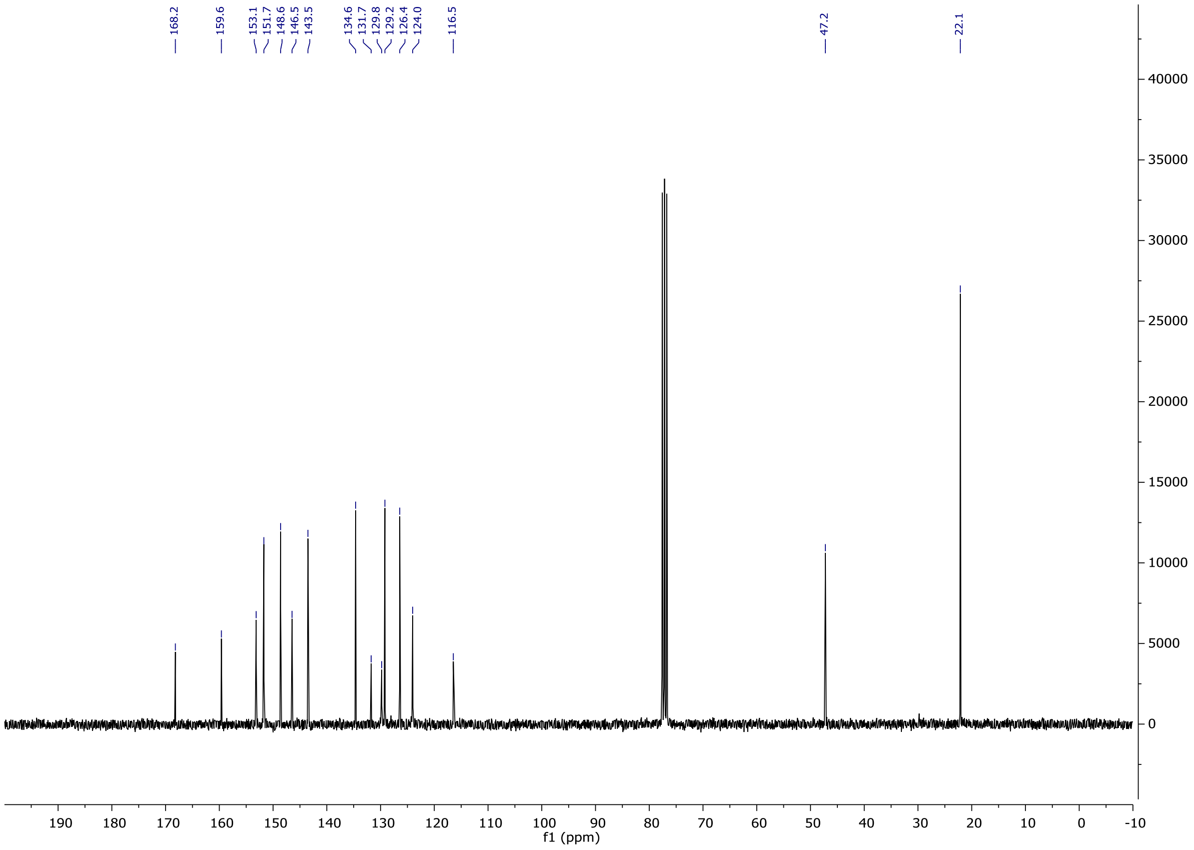

8-Cyclopropyl-2-(pyridin-3-yl)thiazolo[5,4-*f*]quinazolin-9(8*H*)-one (**4c**) (**FC162**)

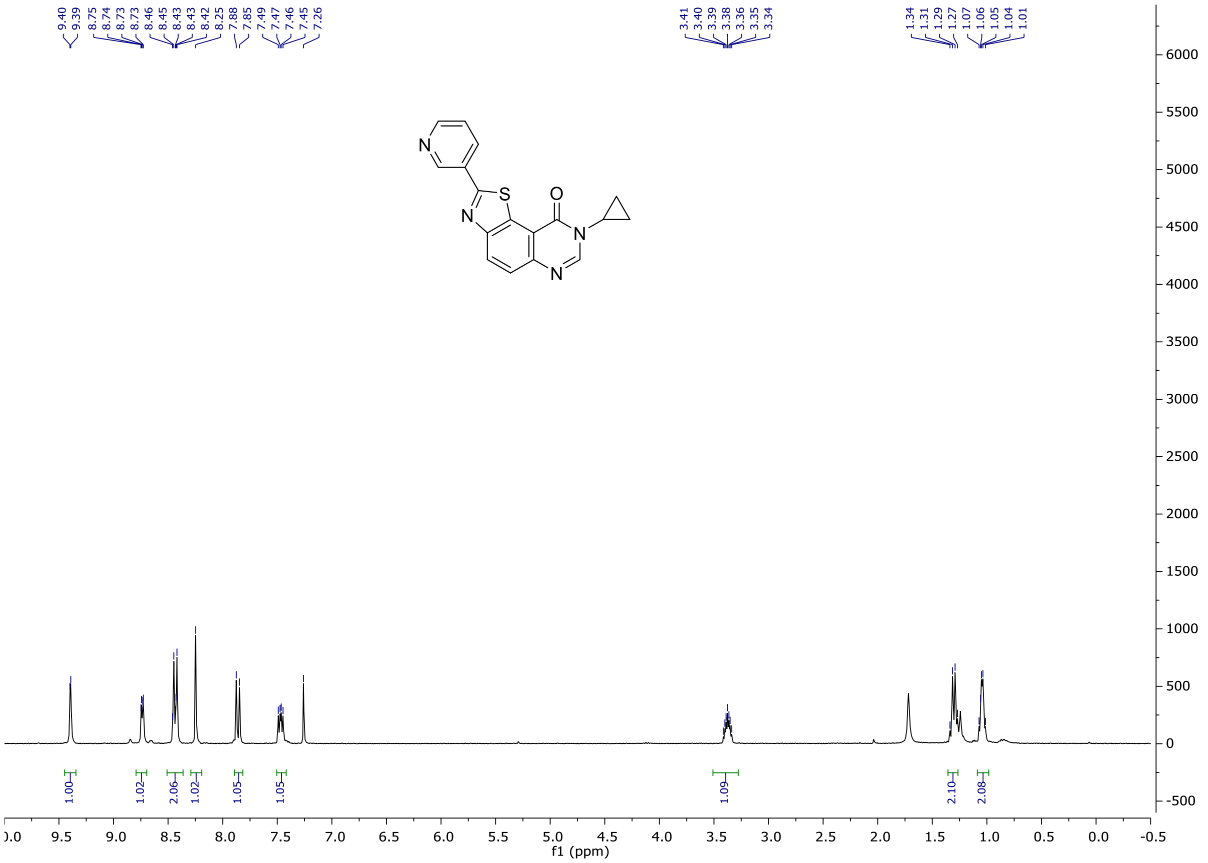

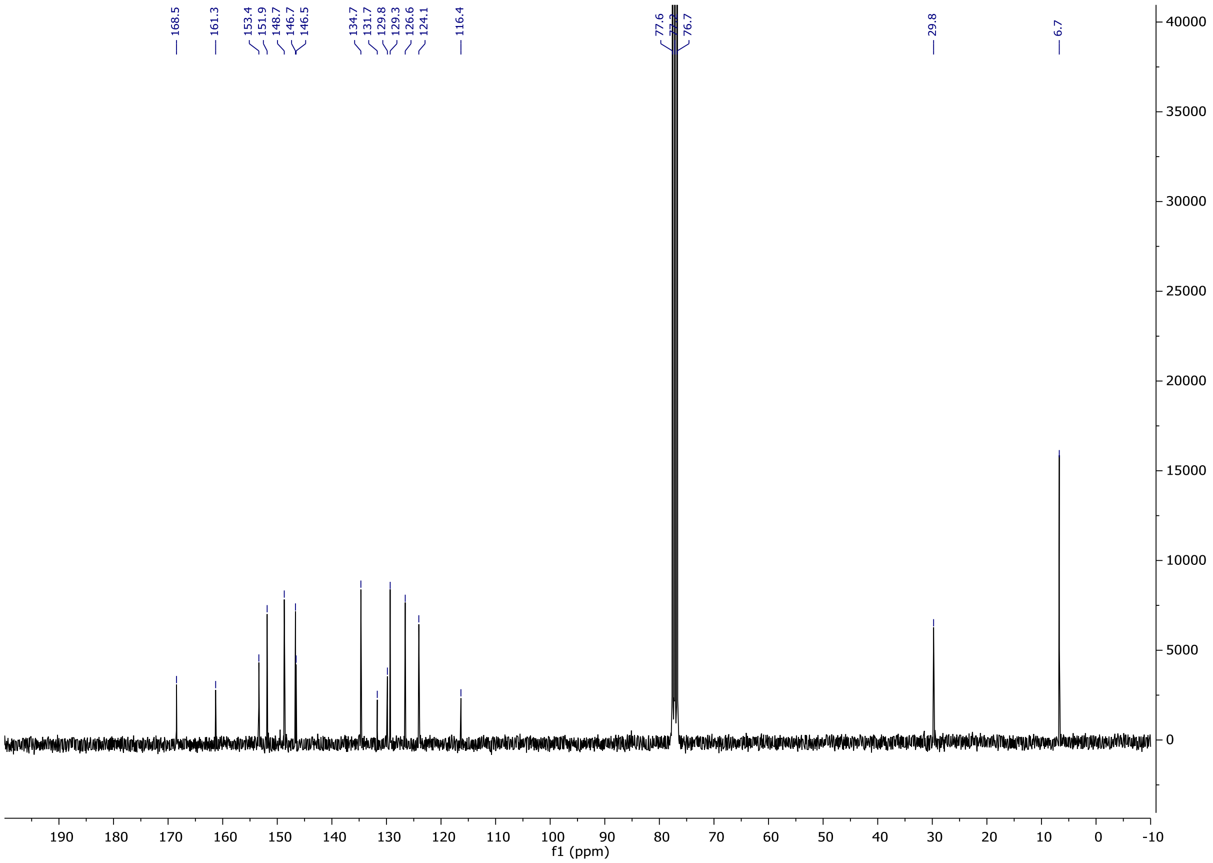

8-Cyclobutyl-2-(pyridin-3-yl)thiazolo[5,4-*f*]quinazolin-9(8*H*)-one (**4d**)

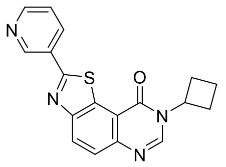

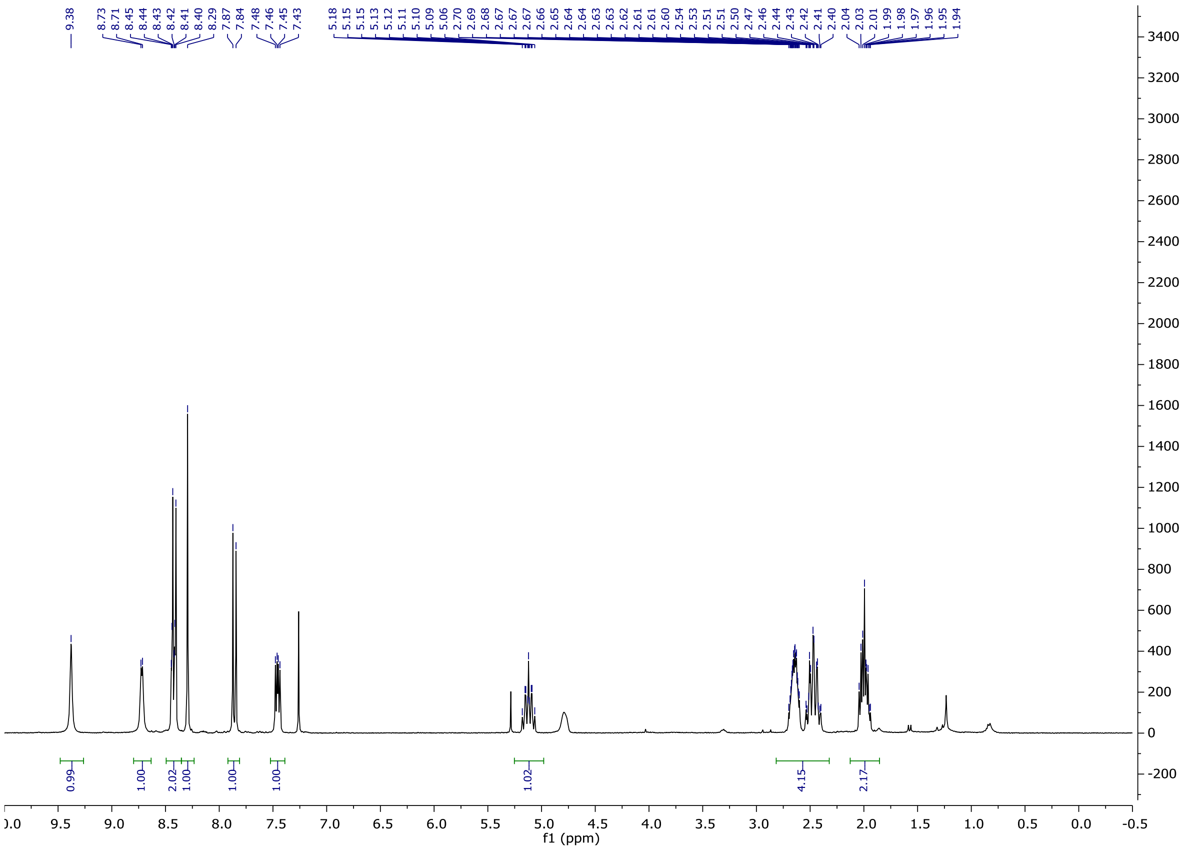

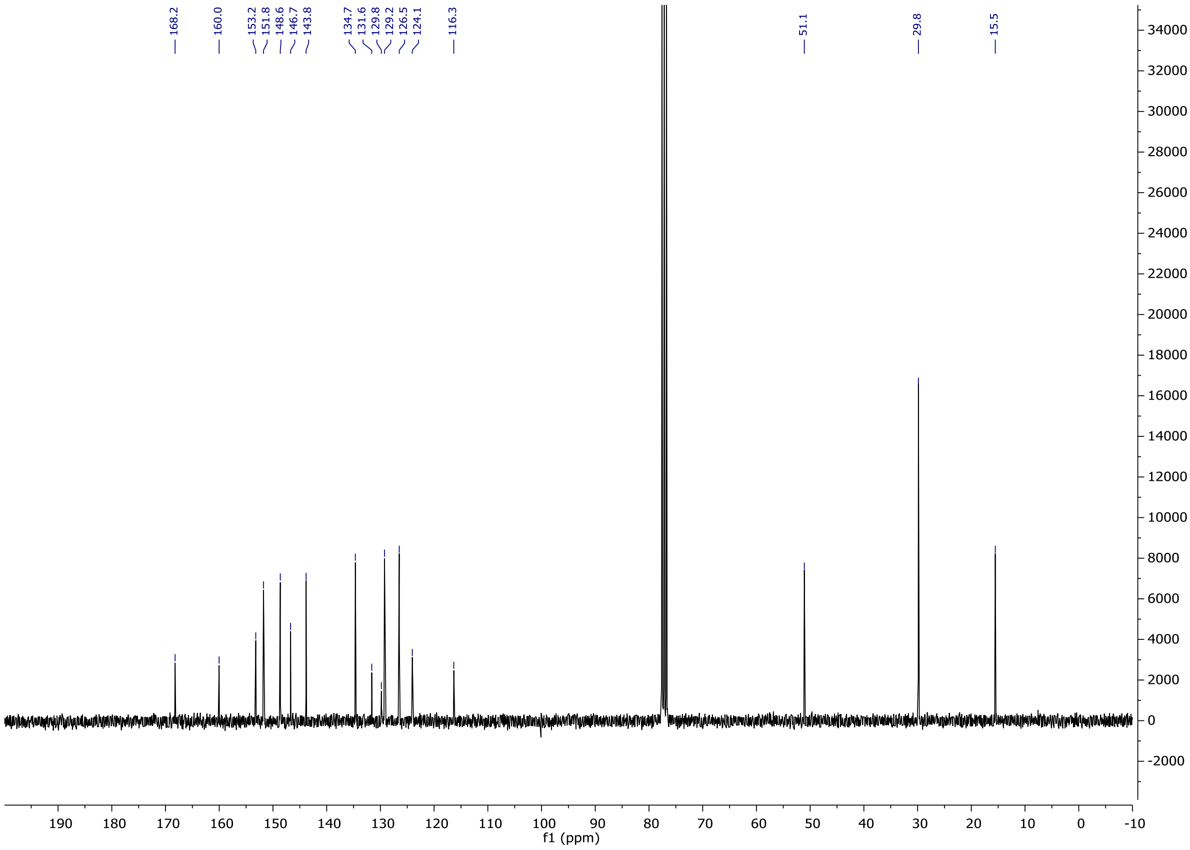

8-Cyclopentyl-2-(pyridin-3-yl)thiazolo[5,4-*f*]quinazolin-9(8*H*)-one (**4e**)

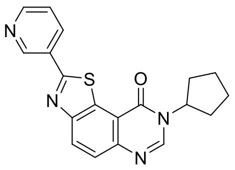

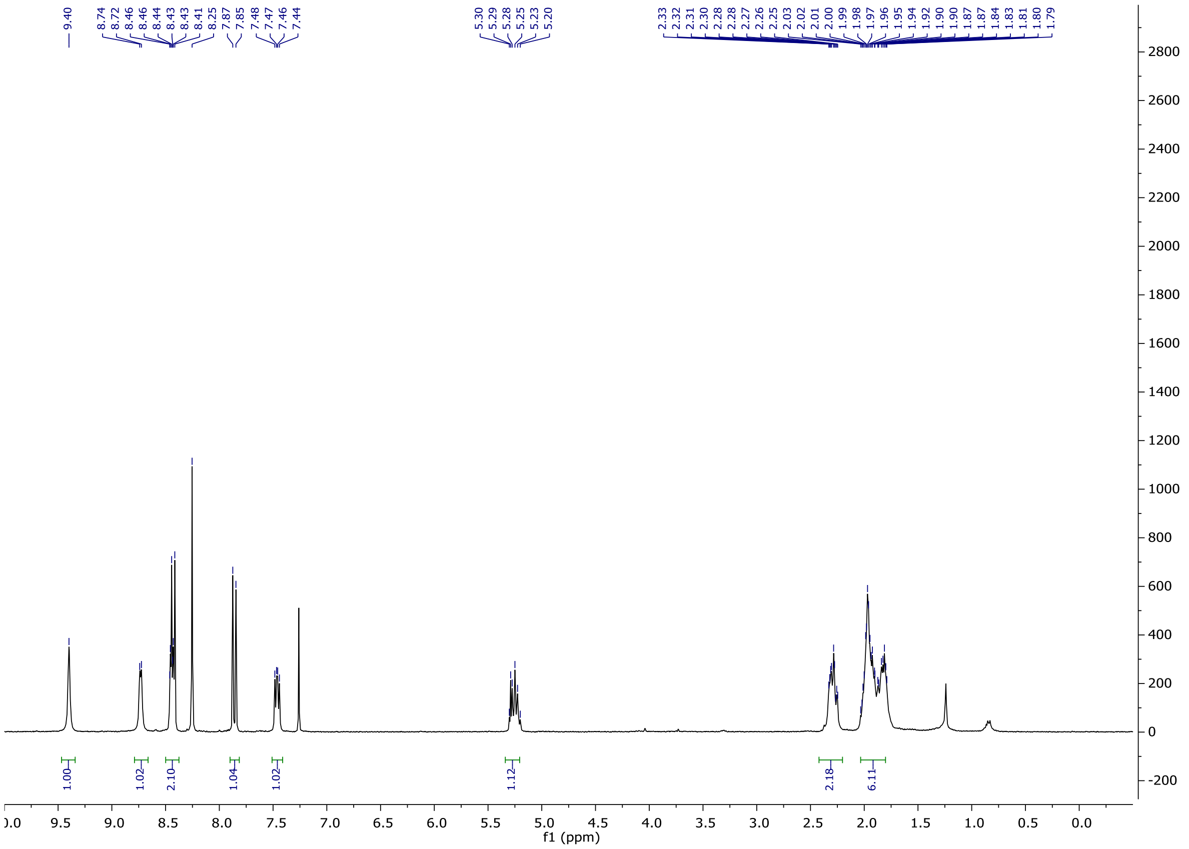

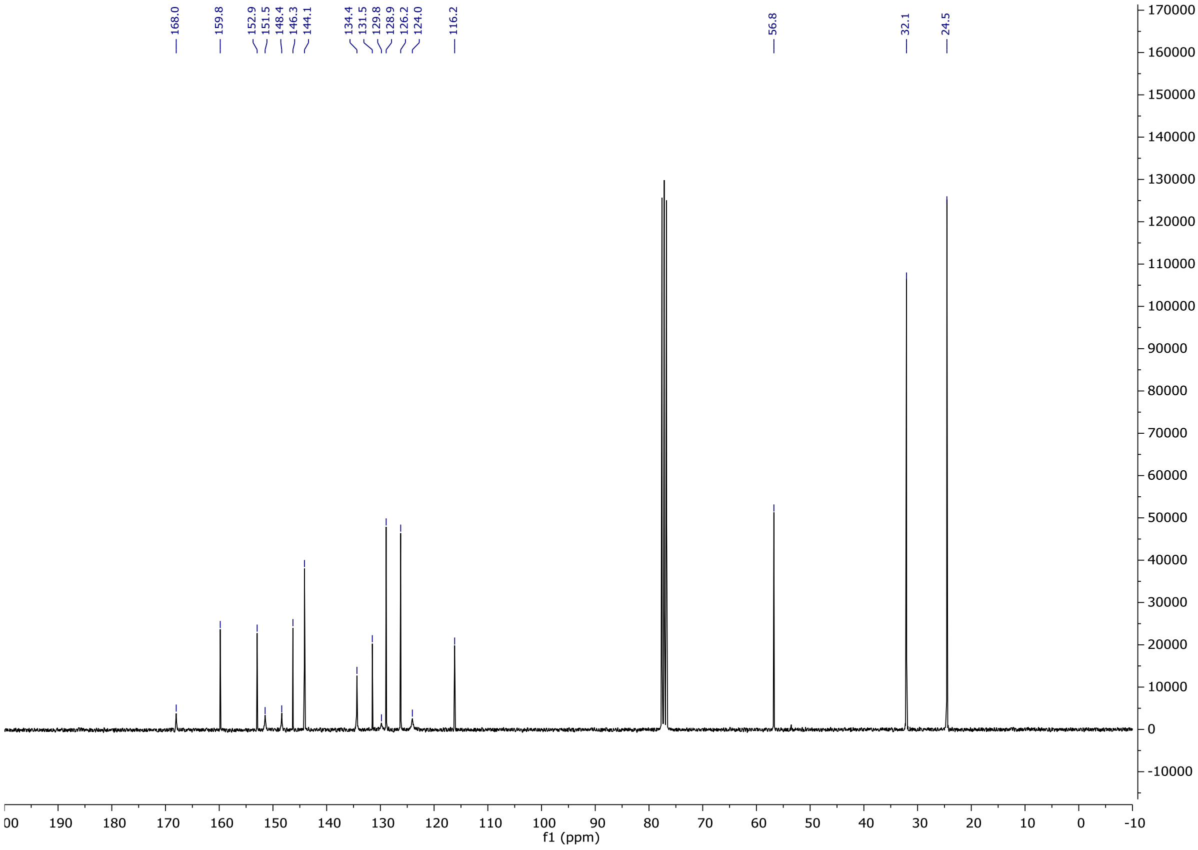

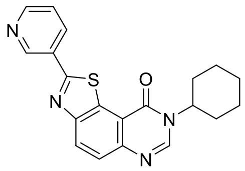

8-Cyclohexyl-2-(pyridin-3-yl)thiazolo[5,4-*f*]quinazolin-9(8*H*)-one (**4f**)

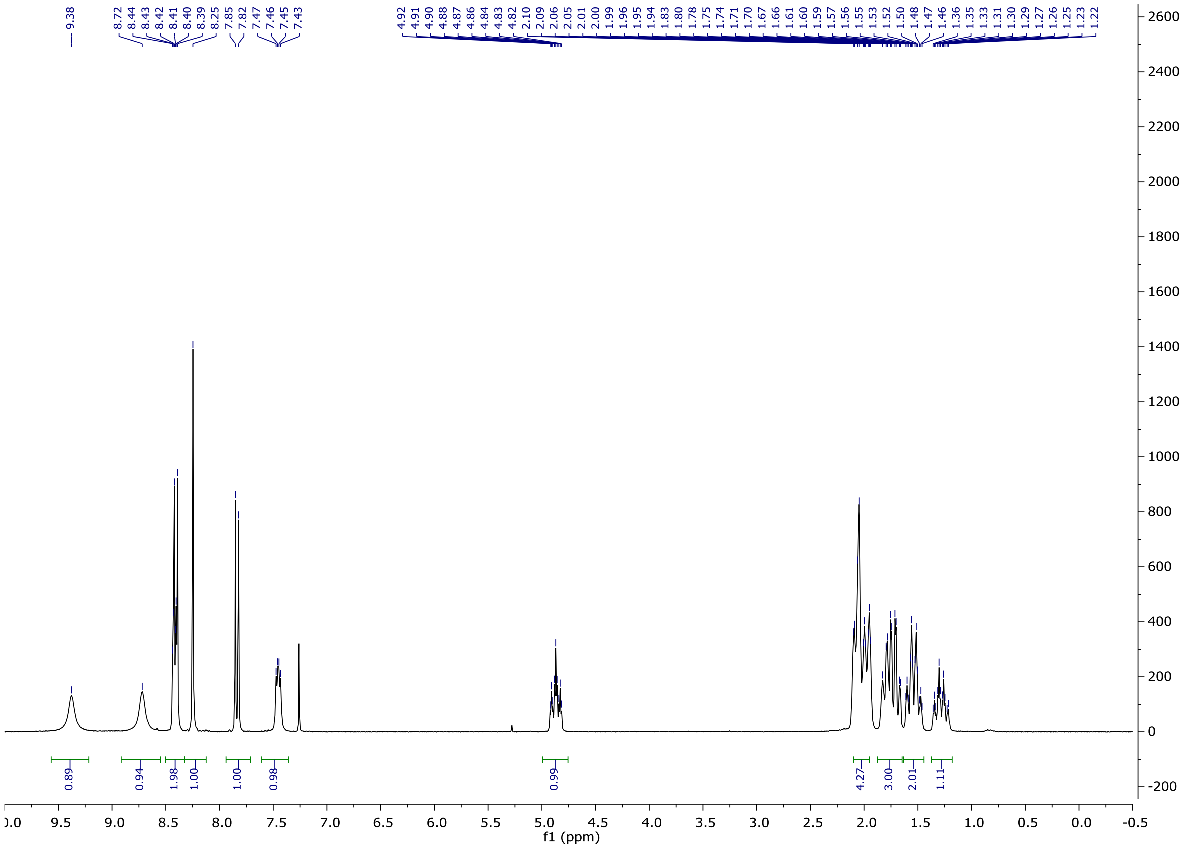

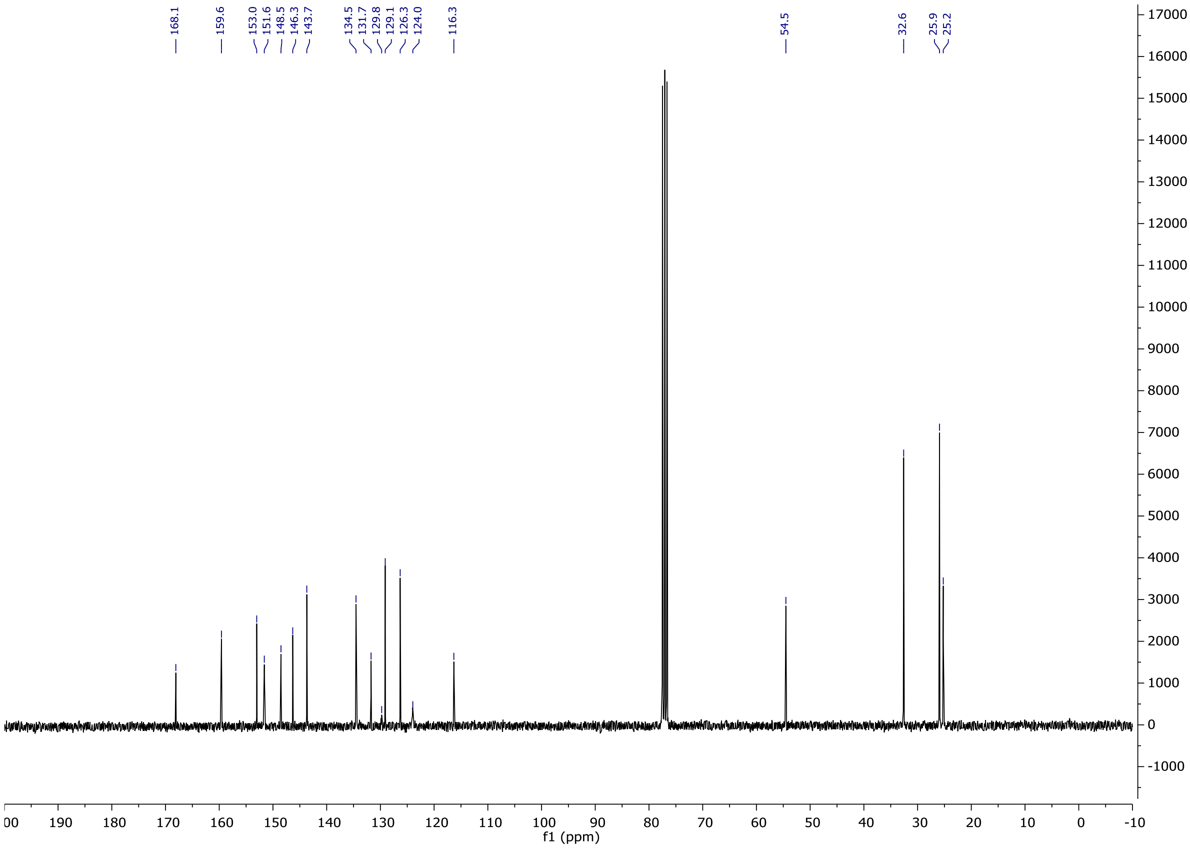

Supplement: Supplementary file 1 [file molecules-23-02181-s001.pdf]
